# Thyroid hormones and metabolic flexibility: tissue-specific control of energy homeostasis

**DOI:** 10.3389/fphys.2026.1882438

**Published:** 2026-06-10

**Authors:** Annunziata Gaetana Cicatiello, Caterina Miro, Serena Sagliocchi, Annarita Nappi, Monica Dentice

**Affiliations:** 1 Department of Clinical Medicine and Surgery, University of Naples “Federico II”, Naples, Italy; 2 CEINGE – Biotecnologie Avanzate S.c.a.r.l., Naples, Italy

**Keywords:** deiodinases, energy homeostasis, exercise, mitochondrial function, thyroid hormones

## Abstract

Thyroid Hormones (THs) are central regulators of energy metabolism, traditionally viewed as key determinants of Basal Metabolic Rate (BMR) and whole-body energy expenditure. Beyond their classical systemic effects, it is now evident that TH action is critically shaped by tissue-specific mechanisms that regulate intracellular hormone availability and signaling. These processes enable a fine-tuned and physiological state-specific control of metabolic activity. In this review, we synthesize current knowledge on the role of THs in energy homeostasis, with a particular focus on the mechanisms that govern local triiodothyronine (T3) signaling. We discuss how the integration of hormone transport, intracellular activation and inactivation by Deiodinases, and receptor-mediated transcriptional responses determine the intensity and timing of TH action in the target tissues. This spatiotemporal regulation supports metabolic flexibility, enabling coordinated control of substrate utilization, mitochondrial function, and energy expenditure under diverse physiological conditions. We further examine how TH signaling is modulated in response to key metabolic challenges, including physical exercise, changes in body weight and adiposity, and circadian rhythms. During exercise, local activation of TH signaling, particularly through type 2 Deiodinase (D2), facilitates mitochondrial adaptation, muscle remodeling, and improved metabolic efficiency. In the context of obesity, interactions between THs and adipose-derived signals such as leptin influence both central and peripheral pathways controlling energy balance, although these mechanisms may become impaired in leptin-resistant states. In parallel, circadian regulation of the Hypothalamic-Pituitary-Thyroid (HPT) axis and peripheral TH metabolism introduces an additional temporal dimension to metabolic control, allowing local tuning of TH action across the day. By integrating molecular, physiological, and systemic perspectives, this review highlights the concept of THs as context-dependent modulators of metabolic flexibility and underscores their potential as targets for interventions in metabolic disorders.

## 1 Introduction

Thyroid Hormones (THs) are fundamental regulators of growth, development, and metabolic homeostasis, coordinating cellular and systemic energy balance in multiple physiological contexts. Among their major functions, THs regulate energy metabolism by acting as key determinants of Basal Metabolic Rate (BMR) and exerting a broad influence on whole-body energy expenditure (Cicatiello et al., 2018). Their metabolic effects encompass multiple pathways, including caloric intake, lipid mobilization and oxidation, cholesterol turnover, gluconeogenesis, and glycolysis (Sinha and Yen, 2024).

Classically, THs are viewed as stimulators of metabolism because they promote energy dissipation through increased mitochondrial activity, ATP turnover, and thermogenesis. At the organ level, THs enhance metabolic activity in tissues that substantially contribute to resting energy expenditure, including the liver, Skeletal Muscle (SkM), heart, and adipose depots. This apparent duality underscores a central role for THs in metabolic flexibility, referred as the ability of cells and tissues to dynamically adjust substrate utilization and energy production in response to changes in nutrient availability, energy demand, and hormonal signals. In the liver, THs stimulate gluconeogenesis and ketogenesis, thereby supplying energy substrates to peripheral tissues such as brain and SkM (Rodd et al., 1992). At the same time, they promote fatty acid β-oxidation and mitochondrial respiration, processes that increase oxygen consumption, while also facilitating *de novo* lipogenesis and triglyceride synthesis when carbohydrate supply is abundant (Sinha et al., 2018; Ritter et al., 2020). In adipose tissue, THs regulate lipid turnover in White Adipose Tissue (WAT) (Obregon, 2014) and enhance thermogenic capacity in Brown Adipose Tissue (BAT) (Triandafillou et al., 1982) through induction of uncoupling protein 1 (UCP1), thereby increasing heat production and contributing to energy expenditure (Cicatiello et al., 2024). In SkM, THs stimulate glucose uptake and glycolytic flux, promote shifts in fiber-type composition toward faster glycolytic phenotypes, and modulate mitochondrial content and function, collectively influencing basal ATP demand (Sagliocchi et al., 2019; Cicatiello et al., 2022; Miro et al., 2023b; Nappi et al., 2025). Moreover, by controlling the expression of the Sarco-Endoplasmic Reticulum Calcium ATPase (SERCA) and the Sodium-Potassium (Na^+^-K^+^) pumps and K^+^-channels, THs are regulators of muscle contraction, excitation-contraction coupling, and energetic consumption (Horowitz et al., 1990; Simonides et al., 1990). In the heart, THs regulate myocardial structure and contractility while sustaining mitochondrial biogenesis and oxidative metabolism, thereby contributing to systemic metabolic rate (Razvi et al., 2018; Murolo et al., 2022).

The metabolic effects of THs emerge from coordinated actions in multiple organs and are strongly influenced by nutritional and hormonal cues. During energy deprivation, suppression of the Hypothalamic-Pituitary-Thyroid (HPT) axis lowers circulating TH levels and decreases BMR as an adaptive response aimed at preserving energy (Sinha and Yen, 2024). In contrast, nutrient availability and adiposity-derived signals such as leptin stimulate Thyrotropin-Releasing Hormone (TRH) production and support TH secretion, thereby sustaining energy expenditure (Lartey et al., 2015; Miro et al., 2025b). TH-dependent control of metabolic rate is therefore dynamic: it is attenuated during caloric restriction to reduce energy consumption, whereas during caloric excess it remains engaged to facilitate carbon source handling, energy partitioning, and redistribution of surplus fuels.

Mechanistically, the metabolic effects of THs are mediated by both genomic and non-genomic pathways. The classical genomic view is based on the premise that triiodothyronine (T3) bound to TH Receptors (TRs) is the principal driver of TH-dependent transcriptional responses (Bianco et al., 2019). At the same time, extranuclear, non-genomic actions of THs have been reported in several cell types and appear to mediate rapid effects through interactions with distinct TH-binding partner proteins (Davis et al., 2018; Nappi et al., 2021). Together, these pathways support the view that TH action is both spatially and temporally regulated, and that its metabolic consequences depend on more than circulating hormone concentrations alone.

In this review, we synthesize current knowledge on the role of THs as regulators of metabolism, integrating systemic and peripheral mechanisms of action. While several excellent reviews have addressed TH signaling and metabolism (Muller and Heuer, 2014; Salvatore et al., 2014; Cicatiello et al., 2018), this work distinguishes itself by proposing an integrative and context-dependent framework in which intracellular TH regulation, through transporters, deiodinases, and receptors, is functionally linked to metabolic flexibility in distinct physiological states, including exercise, obesity, and circadian timing.

We propose that the metabolic effects of THs are best understood within a framework of state-dependent regulation of intracellular hormone availability and signaling, in which metabolic flexibility is governed less by circulating TH levels and more by tissue-specific regulation of intracellular T3 signaling. Accordingly, we discuss how TH action is modulated across physiological and pathophysiological states, including exercise, obesity, and circadian rhythms, with particular emphasis on mechanisms that govern metabolic flexibility.

**Figure 1:**
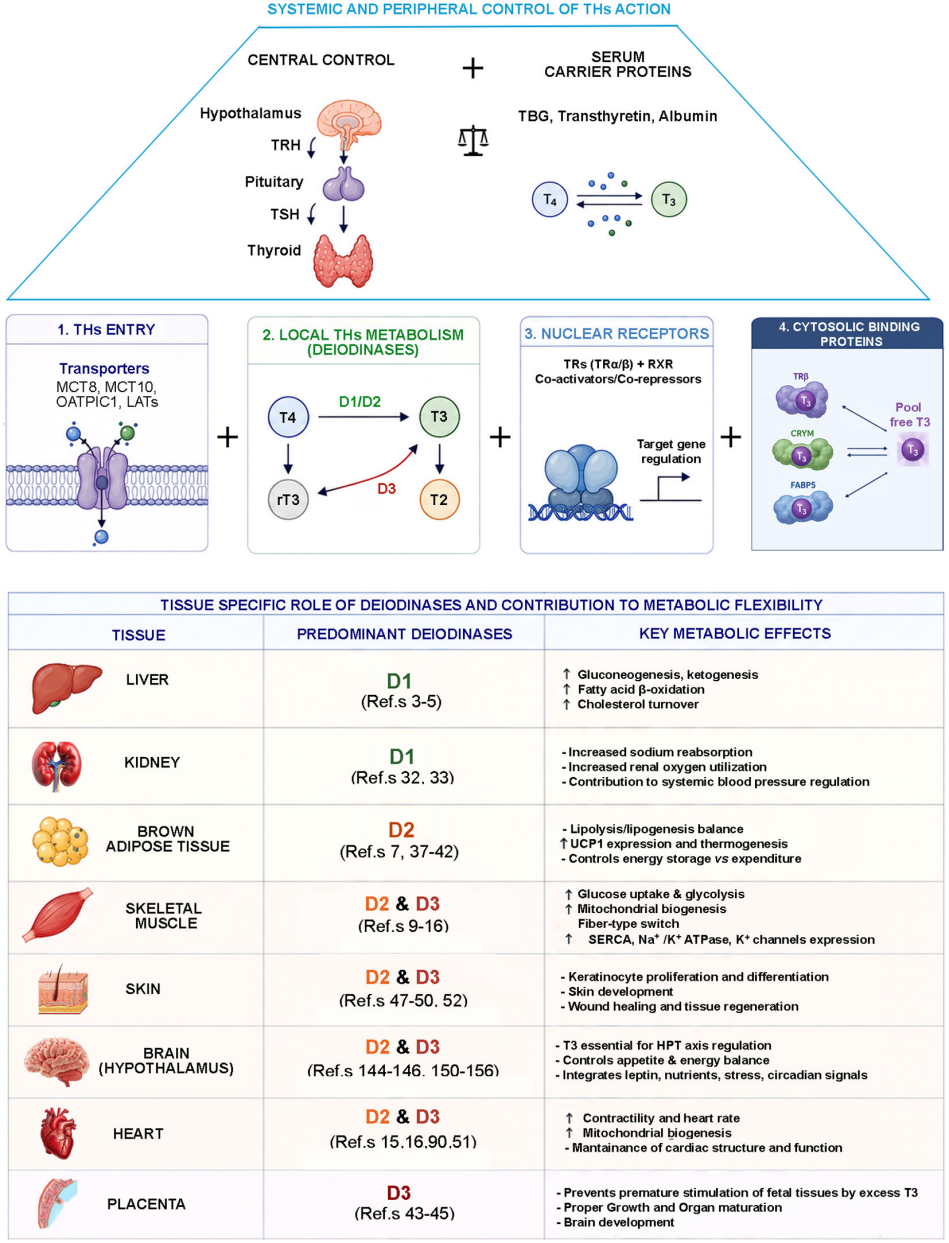
Multilevel regulation of thyroid hormone action and tissue-specific roles of Deiodinases in metabolic flexibility. Schematic overview of systemic and intracellular mechanisms regulating thyroid hormone signaling and metabolism. At the systemic level, TH action is controlled by the hypothalamic–pituitary–thyroid (HPT) axis, including TRH and TSH secretion, together with circulating carrier proteins such as thyroxine-binding globulin (TBG), transthyretin, and albumin, which regulate the transport and bioavailability of thyroxine (T4) and triiodothyronine (T3). Cellular TH action is further modulated by membrane transporters (MCT8, MCT10, OATP1C1, and LATs), intracellular deiodinases (D1, D2, and D3) that activate or inactivate THs, nuclear thyroid hormone receptors (TRα/β) transcriptional regulation, and cytosolic TH-binding proteins that control free T3 intracellular pool. The lower panel summarizes the tissue-specific distribution of deiodinases and the major metabolic effects of TH signaling in liver, kidney, brown adipose tissue, skeletal muscle, skin, brain, heart, and placenta, highlighting the contribution of local TH metabolism to metabolic flexibility and organ-specific physiology.

To integrate the concepts discussed throughout this review, we propose a unifying schematic model in which thyroid hormone transport, intracellular activation by deiodinases, and receptor-mediated signaling are hierarchically organized to drive context-specific metabolic outputs across physiological conditions such as exercise, obesity, and circadian timing (**Figure 1**).

## 2 Spatiotemporal control of intracellular thyroid hormone signaling

The metabolic actions of THs are critically determined by the regulation of intracellular hormone availability within specific tissues. Thus, systemic TH production must be considered alongside local regulatory mechanisms that fine-tune hormone levels and signaling within the intracellular milieu. While the thyroid gland, under the control of the HPT axis and iodide availability, determines circulating concentrations of thyroxine (T4) and T3, peripheral TH action depends on the coordinated activity of iodothyronine Deiodinases, membrane transporters, and TRs.

In 2019, Bianco and colleagues formalized this concept in the “TH action TRIAD,” which integrates transmembrane transport, intracellular deiodination, and receptor engagement as the three major regulatory nodes governing TH signaling at the tissue level (Bianco et al., 2019). This framework emphasizes that TH biology is not simply a function of serum hormone levels, but rather a spatially and temporally organized process in which hormone access, local activation or inactivation, and transcriptional decoding are coordinated to match tissue-specific physiological needs. In metabolic terms, this organization allows different organs to calibrate intracellular T3 signaling according to developmental stage, nutritional state, environmental inputs, and energy demand, allowing energy allocation resilience (Schuler et al., 2026) (**Figure 1**).

### 2.1 Deiodinases as tissue-specific rheostats of intracellular T3 and metabolic adaptation

Deiodinases are selenocysteine-containing enzymes that catalyze the activation and inactivation of THs and occupy a central position in the metabolic regulation of TH action (Dentice et al., 2013; Luongo et al., 2019). The three isoforms, type 1 (D1), type 2 (D2), and type 3 (D3) Deiodinases, are differentially distributed among tissues and exert distinct effects on intracellular hormone signaling (Gereben et al., 2015; Schweizer and Steegborn, 2015). D1 and D2 catalyze the outer-ring deiodination of T4 to produce bioactive T3, thereby amplifying TH signaling independently of circulating hormone levels. By contrast, D3 terminates TH action by converting T4 to reverse T3 (rT3) and T3 to 3,3′-diiodothyronine (T2), thereby attenuating T3-dependent transcriptional responses (Dentice et al., 2013). Through this reciprocal organization, Deiodinases act as local rheostats that adapt intracellular T3 availability to developmental, environmental, and metabolic cues (Miro et al., 2025a) (**Figure 1**).

Deiodinase expression is highly organ-specific and temporally regulated, providing precise control over the intensity and timing of TH signaling. Their activity depends on selenocysteine incorporation, a process mediated by Selenocysteine (SECIS) insertion Sequence-Binding Protein 2 (SBP2). Mutations in SBP2 impair Deiodinase function and result in multisystem phenotypes that include altered TH metabolism, growth retardation, neurodevelopmental defects, and infertility, underscoring the physiological importance of Deiodinase-dependent TH activation (Dumitrescu et al., 2010; Fu et al., 2017).

Among the three isoforms, D1 is predominantly expressed in liver, kidney, and thyroid, where it contributes substantially to circulating T3 production. Because D1 is localized in the plasma membrane, it is well positioned to exchange hormone metabolites with the bloodstream and thus to influence systemic TH homeostasis (Russo et al., 2021; Galton and Hernandez, 2023). In addition, D1 has been proposed to act as an iodide scavenger, facilitating the recovery of iodide from iodothyronine metabolites and contributing to the maintenance of whole-body iodine balance (Luongo et al., 2019). Pharmacological inhibition of D1 by propylthiouracil or high iodide exposure, as well as genetic variants affecting its function, leads to elevated serum rT3 and altered T3/T4 ratios (Luongo et al., 2019). Collectively, these findings indicate that D1 predominantly contributes to shaping the circulating TH milieu and maintaining systemic iodide balance through its scavenger function, whereas its role in fine-tuning intracellular metabolic signaling is comparatively limited relative to D2.

D2, in contrast, is a major determinant of intracellular T3 production and metabolic regulation. Located in the endoplasmic reticulum, D2 generates T3 within cells and thereby directly influences nuclear receptor occupancy and downstream gene expression independently of plasma hormone levels (Bianco, 2013). D2 is highly expressed in metabolically active tissues, including BAT, SkM, brain, and bone, where it supports specific functions linked to energy expenditure and metabolites utilization. Unlike D1, D2 is relatively insensitive to classical antithyroid drugs. Although pharmacological modulation of D2 activity has been explored, the lack of selective and clinically applicable inhibitors currently limits the ability to specifically target D2-dependent TH signaling *in vivo* (Sagliocchi et al., 2023; Acampora et al., 2025). This idea is especially relevant in metabolic disease, where tissue-restricted manipulation of intracellular T3 could potentially improve energy balance without provoking cardiac or skeletal complications.

A paradigmatic example of the metabolic relevance of D2 is cold-induced thermogenesis. Cold exposure activates the sympathetic nervous system and stimulates D2 expression in BAT through cyclic Adenosine Mono-Phosphate (cAMP)-dependent pathways (Bianco and Silva, 1988). This leads to a rapid rise in local T3 production, increased TR occupancy, and induction of thermogenic genes such as UCP1. In this context, D2-mediated T3 generation is indispensable for full activation of BAT metabolism. Indeed, although circulating T3 contributes to thermogenic competence, the locally produced T3 pool is required for maximal induction of the thermogenic program and for coordinated regulation of genes involved in mitochondrial activity, lipogenesis, and fuel utilization (Bianco and Silva, 1987; Bianco and Silva, 1988; Carvalho et al., 1991; Bianco et al., 1992; Bianco et al., 1998; Branco et al., 1999) (**Figure 1**).

Beyond BAT, D2 contributes to metabolic regulation in additional tissues, including SkM, lung, and the Central Nervous System (CNS). Its expression changes during development and in response to metabolic stimuli, allowing temporally appropriate activation of TH signaling. During embryogenesis, D2 and D3 expression establish local T3 gradients that are essential for normal organ development. In the human brain, differential expression of these enzymes in distinct regions generates marked differences in local T3 availability, which are critical for neurodevelopmental processes (Kester et al., 2004). Similar developmental surges in D2 activity have been reported in cochlea and liver, reinforcing the broader principle that local T3 generation is required for the coordinated maturation of metabolically active tissues (Campos-Barros et al., 2000; Fonseca et al., 2015) (**Figure 1**).

In contrast, D3 functions primarily as a negative regulator of TH action. It is prominently expressed during development and is often reactivated in pathological contexts associated with proliferation, remodeling, or reduced metabolic demand. In tumors, D3 overexpression can induce consumptive hypothyroidism, a condition in which excessive TH inactivation necessitates high-dose hormone replacement (Aw et al., 2014). Beyond this systemic effect, D3 is frequently upregulated in diverse malignancies, including cutaneous tumors such as Basal Cell Carcinoma (BCC) (Di Girolamo et al., 2016; Di Girolamo et al., 2026) and Squamous Cell Carcinoma (SCC) (Miro et al., 2019; Miro et al., 2025a), where it is regulated by p53 (Nappi et al., 2023) and contributes to hyperproliferative programs. In hypertrophic myocardium, increased D3 activity reduces local T3 availability and contributes to altered cardiac metabolism (Paolino et al., 2017). In the skin, D3 induction by Hedgehog signaling reduces TH action and favors keratinocyte proliferation through de-repression of T3-sensitive genes such as Cyclin D1 (Dentice et al., 2007). These examples illustrate that D3 is not simply a passive inactivating enzyme but an active participant in the remodeling of local metabolic and proliferative programs.

The reciprocal regulation of D2 and D3 is particularly important in the hypothalamus, where local TH signaling influences feeding behavior, energy expenditure, neuroendocrine function, and adaptive responses to nutritional state. Fasting increases hypothalamic D2 activity and may contribute to suppression of Thyrotropin-Releasing Hormone (TRH) expression, linking energy status to HPT axis regulation (Coppola et al., 2005). Conversely, changes in hypothalamic Deiodinase expression have been implicated in torpor and photoperiod-dependent metabolic adaptations (Watanabe et al., 2007; Cubuk et al., 2017). Consistent with this, D3-deficient mice display increased hypothalamic TH signaling together with altered energy expenditure, reduced adiposity, and disrupted circadian rhythms, emphasizing the importance of local TH inactivation in maintaining metabolic homeostasis (Wu et al., 2017). A similar dynamic interplay between D2 and D3 has been described in cancer, where temporally regulated expression patterns have been reported, with D3 predominating in early stages of tumorigenesis and D2 becoming more prominent in later stages, suggesting a role for Deiodinase switching in the control of tumor progression (Miro et al., 2019; Torabinejad et al., 2023), as well as in the SkM regeneration when the myogenic program is characterized by an initial surge of D3 that sustains muscle cell proliferation and amplification, followed by a rise in D2 expression that promotes the T3-dependent myogenic differentiation (Ambrosio et al., 2013; Dentice et al., 2013; Dentice et al., 2014).

The metabolic relevance of Deiodinases in humans is also supported by genetic studies. Single-Nucleotide Polymorphisms (SNPs) in Deiodinases genes (*DIOs*) can perturb intracellular TH signaling and have been associated with metabolic phenotypes (Bianco and Kim, 2018). The best studied example is the common Thr92Ala variant of *DIO2*, present in approximately 12-36% of the population (Dora et al., 2010). This variant has been linked to traits suggestive of reduced TH action at the tissue level, including insulin resistance, type 2 diabetes mellitus, and hypertension (Mentuccia et al., 2002; Canani et al., 2005; Gumieniak et al., 2007). Mechanistically, Thr92Ala appears to impair D2 catalytic efficiency and/or intracellular processing, thereby reducing T3 generation in D2-expressing tissues such as SkM, adipose tissue, and brain (Castagna et al., 2017; Jo et al., 2019). Mouse studies further support the idea that this variant can attenuate local TH signaling.

The metabolic impact of Thr92Ala-D2 may also depend on interaction with other genetic backgrounds. For example, individuals carrying both Thr92Ala-D2 and the Trp64Arg polymorphism in the β3-adrenergic receptor, which reduces cAMP generation, exhibit increased body mass index (Mentuccia et al., 2002) (Kimura et al., 2000). This observation suggests that impaired local TH activation may synergize with attenuated adrenergic signaling to influence body weight and energy expenditure.

However, these findings have not been consistently replicated across populations, raising concerns regarding their generalizability. Notably, a large cohort study involving more than 12,500 individuals found no association between the Thr92Ala D2 polymorphism and altered thyroid parameters, reduced quality of life, or metabolic syndrome, either in the general population or among individuals receiving TH replacement therapy (Wouters et al., 2017). This inconsistency likely reflects the complexity and redundancy of TH regulation, as well as the contribution of environmental, nutritional, and genetic modifiers. Even so, the available evidence clearly supports the concept that Deiodinases are major determinants of metabolic flexibility, enabling tissues to adjust TH signaling according to developmental stage, environmental challenge, and energy availability.

### 2.2 Coordinated control of hormone access and signal execution: transporters and receptors in metabolic regulation

The initiation of intracellular TH signaling requires regulated transport of hormone across the plasma membrane. Despite their lipophilic nature, THs depend on specific membrane transporters for cellular entry, making transporter expression an important determinant of intracellular hormone action. Monocarboxylate Transporters (MCT8 and MCT10), Organic Anion Transporting Polypeptide 1C1 (OATP1C1), SLC17A4, and L-type Amino Acid Transporters 1 and 2 (LAT1 and LAT2) mediate bidirectional movement of THs across cell membranes (Groeneweg et al., 2020). These transporters preferentially handle the small free fraction of T4 and T3, whereas most circulating hormone remains protein-bound to thyroxine-binding globulin or transthyretin and is therefore not directly available for cellular uptake (Pappa et al., 2015). As a result, intracellular TH availability depends less on total circulating hormone concentrations than on tissue-specific transport capacity (**Figure 1**).

The expression and activity of TH transporters vary substantially among tissues, providing an additional layer of metabolic regulation. By controlling hormone influx and efflux, they shape intracellular TH pools and thereby influence the intensity and duration of T3-dependent transcriptional responses (Friesema et al., 2008; Mayerl et al., 2014). This is particularly important in tissues with high metabolic activity, where precise adjustment of intracellular hormone availability is required to coordinate energy expenditure, substrate selection, and adaptation to nutritional or environmental change.

The metabolic importance of TH transporters is clearly illustrated by genetic defects. Inactivating mutations in MCT8 (encoded by *SLC16A2* gene) cause Allan-Herndon-Dudley syndrome, a severe X-linked disorder characterized by defective TH transport into the brain (Dumitrescu et al., 2004; Friesema et al., 2004). Affected individuals show elevated serum T3, reduced T4, and normal or slightly elevated Thyroid-Stimulating Hormone (TSH) levels, revealing a profound dissociation between circulating hormone levels and intra-tissue TH action. From a metabolic perspective, MCT8 deficiency demonstrates that defective transport creates compartmentalized TH states: some peripheral tissues are exposed to relative T3 excess, whereas the brain experiences profound TH deprivation. This mismatch not only produces severe neurological impairment but also alters systemic metabolic homeostasis by affecting substrate partitioning, tissue metabolism, and energy expenditure.

Animal studies reinforce this concept. Mct8 knockout mice show increased T3 action in liver and reduced signaling in brain, accompanied by dysregulated lipid and glucose metabolism, increased energy expenditure, and reduced adiposity (Di Cosmo et al., 2013). Combined deficiency of *MCT8* and *OATP1C1* more closely reproduces the human phenotype and highlights the cooperative role of transporters in establishing appropriate tissue TH distribution, especially across the blood-brain barrier (van Geest et al., 2021).

Once TH enters the cell, its metabolic effects are ultimately executed by TRs, TRα and TRβ (encoded by *THRA* and *THRB* genes, respectively), which translate intracellular T3 availability into transcriptional responses. TRα and TRβ are expressed from early embryogenesis onward and display tissue-specific patterns that underlie much of the heterogeneity of TH action. TRα predominates in SkM, heart, bone, and BAT, whereas TRβ is highly expressed in liver, pituitary, hypothalamus, and selected regions of the CNS. This differential distribution allows THs to regulate energy expenditure, carbon source metabolism, and endocrine feedback in an organ-specific manner.

TRs signaling is itself subject to regulation. THs can modulate TRs expression in a tissue-dependent fashion, and receptor activity is further shaped by post-translational modifications such as phosphorylation, acetylation, and SUMOylation (Liu and Brent, 2018). These processes affect receptor stability, localization, and transcriptional activity, thereby tuning cellular responsiveness to TH under changing physiological conditions.

The metabolic importance of receptor-mediated signaling is highlighted by Resistance to Thyroid Hormone (RTH) syndromes caused by mutations in *THRA* or *THRB* genes. Mutations in *THRB*, particularly in the ligand-binding domain, impair pituitary negative feedback and lead to elevated circulating TH levels with inappropriately normal or increased TSH (Refetoff et al., 1993). Yet, despite systemic thyrotoxicosis, patients exhibit reduced TH action in TRβ-predominant tissues, including liver and pituitary, and often develop dyslipidemia, insulin resistance, and non-alcoholic fatty liver disease (Chaves et al., 2021). Meanwhile, elevated circulating TH overstimulates TRα-rich tissues, leading to increased BMR, tachycardia, and accelerated bone turnover. This phenotype illustrates how receptor isoform distribution determines the metabolic consequences of abnormal TH signaling.

By contrast, *THRA* mutations primarily impair TRα-mediated responses and produce a phenotype resembling intra-tissue hypothyroidism. Patients often present with reduced BMR, bradycardia, constipation, dyslipidemia, and skeletal and neurodevelopmental abnormalities (Bochukova et al., 2012; Moran and Chatterjee, 2015; Dore et al., 2023). These manifestations are consistent with diminished TH action in muscle, heart, gastrointestinal tract, and brain, further underscoring the importance of TRα in maintaining basal energy expenditure and metabolic tone. Again, circulating TH values are only modestly abnormal, emphasizing that receptor function, rather than serum hormone levels alone, determines metabolic phenotype.

At the molecular level, TR-dependent metabolic regulation extends beyond direct activation of canonical TH-responsive genes. Although some targets contain well-defined TH Response Elements (TREs), the metabolic transcriptional footprint of THs is much broader (Singh et al., 2018b). This amplification likely involves secondary induction of transcription factors, non-coding RNAs, and epigenetic regulators (Aranda, 2021). Particularly relevant examples include induction of Peroxisome Proliferator-activated receptor Gamma Coactivator 1-alpha (PGC-1α) and Estrogen-Related Receptor alpha (ERRα), which promote mitochondrial biogenesis and oxidative metabolism (Singh et al., 2018a), and activation of pathways involving Sirtuin 1 (SIRT1), which further expand TH-dependent control of cellular metabolism (Singh et al., 2013; Thakran et al., 2013). Thus, receptors do not simply transmit TH signals; they also amplify them through broader metabolic transcriptional networks.

Together, transporters and receptors define two tightly linked checkpoints in TH-dependent metabolic regulation: one governs hormone access to the cell, and the other governs transcriptional interpretation of the intracellular T3 signal. Their integration ensures that tissue metabolism responds to local physiological requirements rather than to serum hormone levels alone (**Figure 1**).

### 2.3 Intracellular T3 buffering systems and tissue-specific oscillations in metabolic signaling

T3 binds TRs with an affinity approximately 20-fold greater than T4 and is therefore the principal mediator of genomic TH action, although T4 may exert limited genomic and non-genomic effects (Gil-Ibanez et al., 2017; Davis et al., 2021). Despite extensive study of receptor-mediated signaling, the mechanisms by which T3 traffics from the cytosol to the nucleus remain incompletely understood. A nuclear-cytoplasmic gradient of T3 has been proposed (Oppenheimer and Schwartz, 1985), but direct experimental demonstration is still lacking. In addition, T3 may interact with cytoplasmic TRs and accelerate their nuclear translocation, suggesting that intracellular trafficking itself may influence the timing of transcriptional responses (Grontved et al., 2015).

An additional regulatory layer is provided by low-capacity, high-affinity cytosolic T3-binding proteins (Dillman et al., 1974). Analogous to plasma TH-binding proteins, these intracellular factors establish an equilibrium between bound and free T3 within the cytoplasm. Because only the free fraction is available for nuclear import and receptor binding (Oppenheimer and Schwartz, 1985), intracellular TH signaling depends not simply on total tissue T3 content but on the proportion of hormone that remains bioavailable. This distinction is metabolically important, as it implies that tissues with similar total T3 concentrations may differ substantially in effective TH signaling depending on their intracellular buffering capacity (**Figure 1**).

This concept is especially relevant for tissues such as liver and kidney, which contain relatively high levels of intracellular T3 together with specific binding proteins, including μ-crystallin (CRYM) in liver (Kinney and Bloch, 2021) and NADPH-dependent Cytosolic T3-Binding Protein (CTBP) in kidney (Hashizume et al., 1989). These proteins may act as intracellular reservoirs that stabilize T3 and modulate the kinetics of its release. In principle, such buffering systems could generate intracellular oscillations in free T3 availability and thereby shape the amplitude and duration of TH signaling in response to metabolic cues. This may have important consequences for processes such as gluconeogenesis, lipid metabolism, and mitochondrial function. However, whether these proteins serve only as passive reservoirs or more actively regulate intracellular T3 trafficking remains unresolved.

The physiological relevance of cytosolic T3-binding proteins is supported, albeit indirectly, by human genetics. Rare CRYM mutations that abolish T3 binding have been associated with sensorineural deafness (Oshima et al., 2006; Suzuki et al., 2007; Wang et al., 2020), although a direct causal link to altered TH signaling has not yet been firmly established. Even so, these observations suggest that intracellular T3 buffering may have meaningful organ-specific effects.

Taken together, cytosolic T3-binding proteins may constitute a fourth regulatory layer of TH action, complementing transport, deiodination, and receptor signaling. By modulating the balance between free and bound hormone within the cell, they may influence the spatiotemporal profile of intracellular T3 action and add further precision to TH-dependent metabolic regulation.

## 3 Thyroid hormone and exercise as determinants of metabolic flexibility

**Figure 2:**
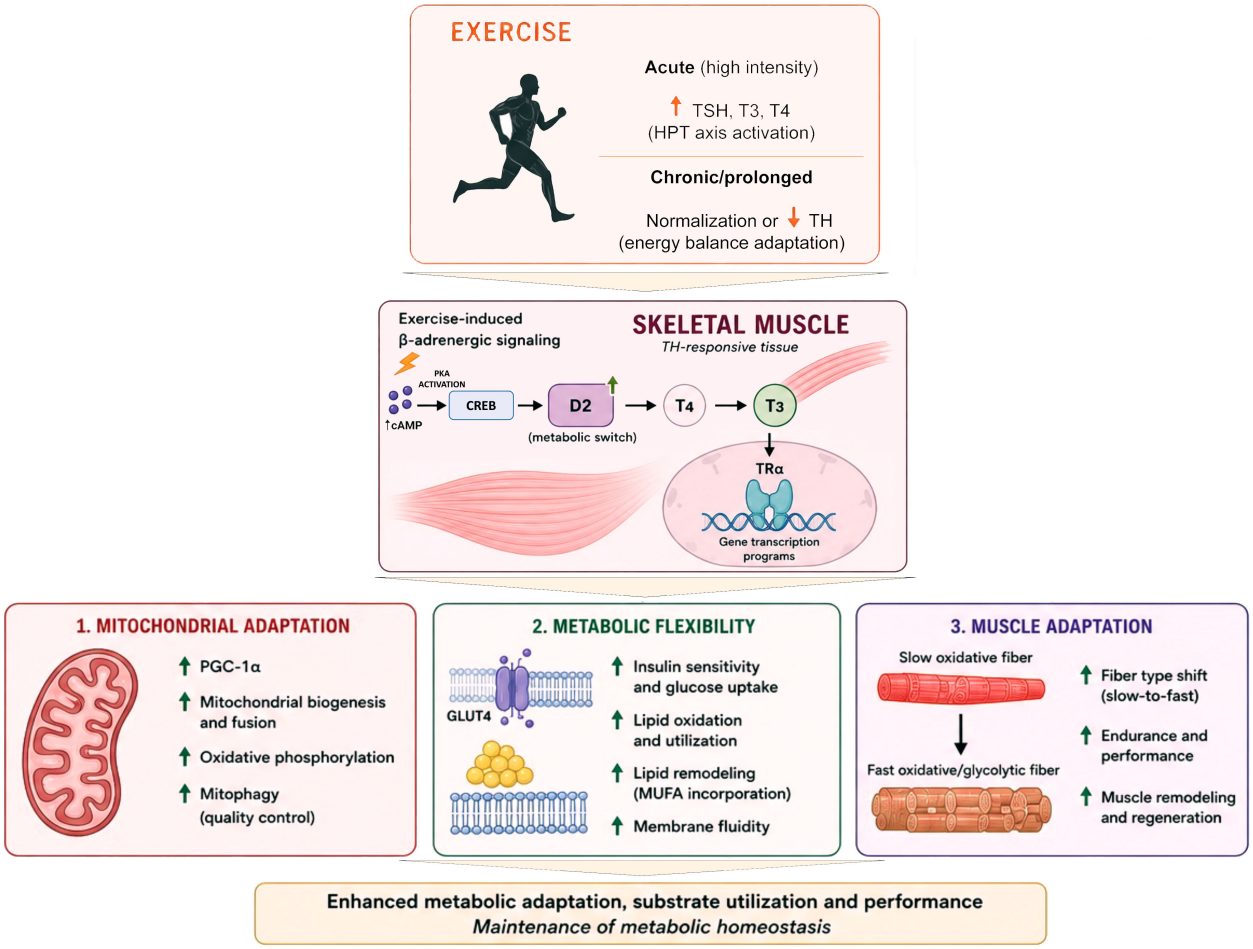
Bidirectional interplay between exercise and thyroid hormones shapes skeletal muscle metabolic adaptation. The figure illustrates the reciprocal relationship between exercise and Thyroid Hormone (TH) signaling and their integrated role in regulating skeletal muscle metabolism. Acute, high-intensity exercise leads to increased circulating Thyroid-Stimulating Hormone (TSH), T4, and T3 levels to support elevated energy demand. In contrast, chronic or prolonged training induces normalization or attenuation of systemic TH levels, preventing excessive hypermetabolism and promoting energy homeostasis. At the SkM level, exercise stimulates β-adrenergic signaling, which induces type 2 Deiodinase (D2), enhancing local conversion of T4 to the active hormone T3. T3, through Thyroid Hormone Receptor α (TRα), drives transcriptional programs that remodel muscle metabolism. These adaptations include (1): mitochondrial biogenesis, dynamics, and quality control (2); improved metabolic flexibility, characterized by increased glucose uptake, lipid oxidation, and membrane remodeling; and (3) structural and functional muscle remodeling, including fiber-type switching toward fast-twitch phenotypes and enhanced performance capacity. Collectively, these coordinated systemic and local responses optimize substrate utilization, enhance metabolic efficiency, and maintain bioenergetic homeostasis during exercise adaptation.

Exercise and THs are tightly interconnected regulators of metabolic flexibility, both shaping energy expenditure, substrate utilization, and adaptive responses to physiological stress. SkM, as a primary site of energy consumption and a major TH-responsive tissue (Simonides and van Hardeveld, 2008), represents a key interface where endocrine and mechanical stimuli converge. The interaction between TH signaling and exercise is bidirectional: exercise modulates systemic and local TH availability, while THs, in turn, determine the efficiency of metabolic adaptation to physical activity. This reciprocal interplay is essential for maintaining metabolic homeostasis and optimizing performance under varying energetic demands (**Figure 2**).

### 3.1 Effects of exercise on circulating TH levels

Physical exercise constitutes a potent physiological stressor that engages the HPT axis, a central component of the thyrotropic multiloop control circuit coordinating endocrine responses to metabolic stress (Petrowski and Kahaly, 2025). Acute exercise, particularly at high intensity activity, transiently increases circulating levels of TSH, T3, and T4, reflecting activation of the HPT axis and a rapid endocrine response aimed at supporting increased energy expenditure (Hermann et al., 2021) (Hermann et al., 2019). In contrast, chronic exercise or prolonged energy deficit is associated with a normalization or reduction of circulating TH levels, representing an adaptive mechanism to preserve energy balance and prevent excessive catabolism (Greenwood and Fleshner, 2011; Mikkelsen et al., 2017). Thus, exercise induces a dynamic modulation of thyroid function, characterized by an initial activation (Gonzalez et al., 1998) followed by recalibration of TH signaling (Mastorakos and Pavlatou, 2005; Uribe et al., 2014). Importantly, these systemic changes do not fully capture the metabolic effects of THs during exercise, which are largely determined by peripheral regulation of intracellular hormone activation, particularly in SkM (**Figure 2**).

### 3.2 Exercise-induced modulation of deiodinase activity

In exercise, a significant increase of T3 serum levels, in presence of unaffected serum T4 levels, is registered due to either an increase in secretion or in peripheral D2-dependent 5′-Deiodinase activity. This regulation of D2 is due to the activation of cellular pathways that converge on *DIO2* gene expression. For example, D2 is positively regulated in muscle by catecholamines (Hosoi et al., 1999), the Protein Kinase A (PKA)-cAMP pathway (da-Silva et al., 2007; Grozovsky et al., 2009), polyphenols and proteasome inhibitors, bile acids (Watanabe et al., 2006), insulin and insulin-sensitizing agents (Grozovsky et al., 2009); and negatively regulated by its own natural substrate, T4, and Tumor Necrosis Factor-α (TNF-α) (Hosoi et al., 1999). Acute, high-intensity exercise increases D2 activity and D2 mRNA expression in SkM through β-adrenergic signaling, linking sympathetic activation to local TH activation. This transient increase in D2-mediated T3 production is essential for initiating adaptive transcriptional programs required for muscle remodeling and metabolic adaptation. Genetic models have demonstrated the functional importance of this pathway: muscle-specific disruption of D2 impairs exercise-induced adaptations (Bocco et al., 2016) and compromises muscle regeneration (Dentice et al., 2010), highlighting the necessity of locally generated T3 for proper muscle function. These findings support the concept that D2 acts as a temporal “metabolic switch,” enabling SkM to rapidly enhance TH signaling when increased metabolic output is required (**Figure 2**).

### 3.3 Role of TH in mitochondrial adaptation and endurance

Mitochondria are central regulators of cellular bioenergetics and metabolic homeostasis, and their function is tightly controlled by THs (Sagliocchi et al., 2024). Through both genomic and non-genomic mechanisms, THs modulate mitochondrial activity, influencing oxidative phosphorylation (Salvatore et al., 2014; Lombardi et al., 2015), substrate utilization (Cicatiello et al., 2022), and Reactive Oxygen Species (ROS) dynamics (Sagliocchi et al., 2019). During exercise, TH-dependent signaling contributes to mitochondrial remodeling and endurance capacity. A key mediator of this process is PGC-1α, a master regulator of mitochondrial biogenesis and oxidative metabolism, whose expression and activity are stimulated by T3 at both transcriptional (Bassett et al., 2003) and post-transcriptional levels (Canto et al., 2009). D2-induced T3 production during exercise contributes to PGC-1α activation, linking local TH activation to mitochondrial expansion and improved aerobic capacity (Bocco et al., 2016). In addition, THs influence mitochondrial efficiency through the regulation of uncoupling proteins, such as UCP3, which modulate energy dissipation and thermogenesis in SkM (Barbe et al., 2001; de Lange et al., 2001; Ramadan et al., 2011). An additional effect of T3 on mitochondria is the switch from slow-to-fast muscle fiber (Simonides and van Hardeveld, 2008), essential to adapt muscle locomotory activity. THs also regulate autophagy, including mitophagy (Grumati et al., 2010), facilitating the removal of damaged mitochondria and maintaining mitochondrial quality during repeated cycles of contraction and recovery (Lo Verso et al., 2014; Lesmana et al., 2016). This integration of mitochondrial biogenesis and turnover is essential for sustaining muscle performance and preventing metabolic dysfunction (**Figure 2**).

### 3.4 TH as mediators of exercise-induced metabolic plasticity

SkM exhibits remarkable metabolic plasticity, allowing rapid adjustments in ATP production and substrate utilization in response to exercise (Lira et al., 2010). THs play a central role in this adaptive capacity by coordinating lipid metabolism, glucose utilization, and membrane composition. TH signaling enhances insulin sensitivity (Pagel-Langenickel et al., 2008) and promotes lipid remodeling in SkM, including increased incorporation of monounsaturated fatty acids such as oleic acid into membrane phospholipids (Miro et al., 2023b). This modification improves membrane fluidity and facilitates glucose transporter translocation, thereby optimizing glucose uptake during exercise. Furthermore, THs induce metabolic reprogramming of muscle fibers, characterized by higher PKM2 expression over the PKM1 isoform (typical of trained Myosin Heavy Chain isoform IIx (MHC-IIx) rich fast fibers) (Verbrugge et al., 2020), and higher LDHA over the LDHB isoform, associated with Oxidative Phosphorylation (OxPhos)-to-glycolysis shift (Liang et al., 2016). Through these mechanisms, THs enable SkM to adapt efficiently to exercise stimuli, enhancing both performance and metabolic resilience (**Figure 2**).

Beyond muscle, the physiological response to exercise involves a broader, integrated network linking thyroid function, the gut microbiome, and mitochondrial activity. This coordinated adaptation can be conceptualized as an allostatic regulation of the thyroid-gut-mitochondrial axis, in which multiple organs dynamically adjust to sustain metabolic balance under exercise-induced stress (Odriozola et al., 2025; Acampora et al., 2026). Within this framework, thyroid and gut functions are closely interconnected. THs regulate key aspects of intestinal physiology, including gut motility, luminal pH, bile acid secretion, and nutrient absorption, thereby contributing to the maintenance of microbial diversity and intestinal homeostasis. Conversely, the gut microbiota actively modulates TH signaling. Through these mechanisms, the microbiome contributes to optimizing substrate utilization, thermogenic capacity, and overall metabolic efficiency during exercise. Importantly, disruption of thyroid status can impair this axis, leading to alterations in gut physiology and microbial composition. Such imbalances may favor the expansion of opportunistic or pathogenic species and promote biofilm formation, ultimately compromising intestinal and metabolic health (Cicatiello et al., 2014; Colicchio et al., 2015; Pagliuca et al., 2016; Picascia et al., 2017).

## 4 The complex relationship between thyroid hormone and body weight

**Figure 3:**
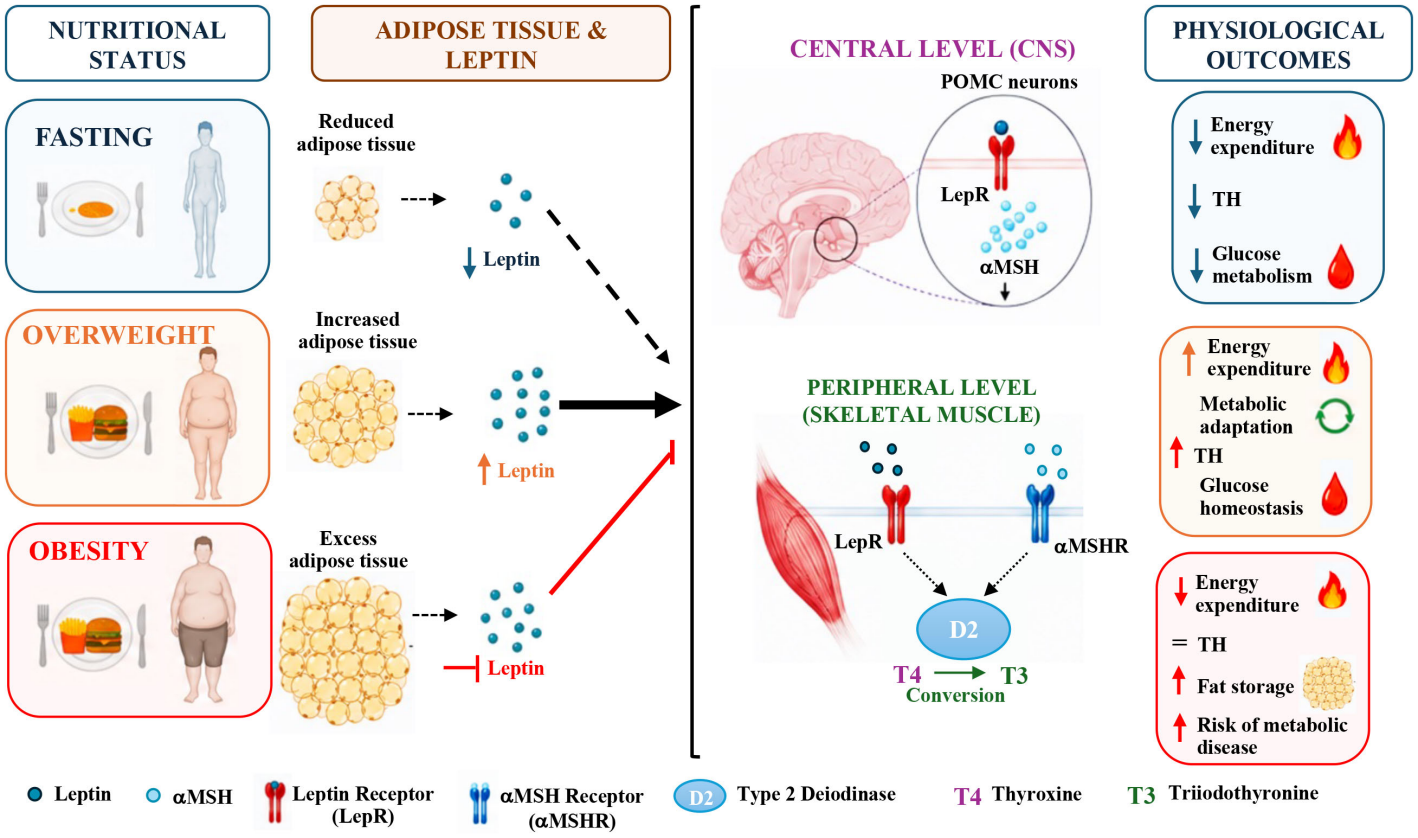
The relationship between thyroid hormones and body weight in the regulation of metabolism. The figure shows how different nutritional conditions impact leptin signaling at the central and peripheral levels. Leptin-TH crosstalk is directly proportional to physiological outcomes. Decreased fat deposits in fasting condition results in reduced leptin release, less activation of Leptin Receptors (LepR) and αMSH Receptors (αMSHR) in the Central Nervous System (CNS) and skeletal muscle (dashed arrow), thus reducing TH action and energy expenditure. In the overweight state, a higher leptin release (bold arrow) elevates TH action as a compensatory mechanism. The excessive fat accumulation in obesity along with the onset of leptin resistance results in ineffective leptin signaling (inhibitory arrow) and an increased risk of metabolic dysregulations.

While exercise highlights acute adaptive flexibility, chronic energy states such as obesity reveal how this system becomes dysregulated. The relationship between THs and body weight is complex and bidirectional, reflecting the integration of endocrine, metabolic, and behavioral pathways that collectively regulate energy balance (**Figure 3**). Although TH excess is typically associated with weight loss and increased energy expenditure and TH deficiency with weight gain and reduced metabolic rate, these associations are not linear (Cicatiello et al., 2018). In particular, alterations in TH signaling are frequently observed in metabolic syndrome, a cluster of conditions including insulin resistance, dyslipidemia, and central obesity, where subtle changes in TH status are linked to impaired metabolic homeostasis. These observations suggest that TH action, rather than circulating hormone levels per se, plays a critical role in the pathophysiology of obesity and related metabolic disorders.

In addition to regulating energy expenditure, THs also influence feeding behavior through central mechanisms. At the hypothalamic level, TH signaling modulates neuropeptide systems involved in appetite control, including the melanocortin pathway, thereby contributing to the integration of energy intake with peripheral metabolic demands (Kim et al., 2014). This central role places THs within a broader neuroendocrine network that coordinates energy homeostasis.

A key component of this network is the crosstalk between adipose tissue and TH signaling, mediated in large part by leptin (Nicholson et al., 2018). Leptin is an adipocyte-derived hormone whose circulating levels reflect fat mass and long-term energy stores (Considine et al., 1996; Chan et al., 2003). Acting as a signal of energy status, leptin regulates both food intake and energy expenditure (Montague et al., 1997). Its absence in humans and animal models results in severe obesity, which can be reversed by leptin replacement, underscoring its fundamental role in energy homeostasis (Pelleymounter et al., 1995; Farooqi et al., 1999). At the central level, leptin acts on hypothalamic neurons expressing the leptin receptor, particularly proopiomelanocortin (POMC) neurons, stimulating the production of α-Melanocyte-Stimulating Hormone (α-MSH), which suppresses appetite and increases energy expenditure (Chen et al., 1996; Chua et al., 1996; Lee et al., 1996; Fan et al., 1997) (**Figure 3**).

Importantly, leptin also interacts with the HPT axis. By stimulating TRH production in the hypothalamus, leptin promotes TSH secretion and supports TH production, thereby linking adiposity to thyroid function (Legradi et al., 1997; Ahima and Flier, 2000; Mullur et al., 2014). This leptin-TSH axis represents a critical mechanism through which energy stores influence systemic TH availability and, consequently, metabolic rate.

Beyond its central actions, leptin exerts direct metabolic effects in peripheral tissues, including SkM, where it intersects with TH signaling at the level of intracellular hormone activation (Miro et al., 2025b). Notably, leptin induces the expression of D2 enzyme responsible for T4-to-T3 conversion within cells (**Figure 3**). This regulation occurs through both direct and indirect mechanisms. Directly, leptin binds to its receptor in SkM and activates the JAK-STAT signaling pathway, leading to transcriptional induction of the *DIO2* gene. Indirectly, leptin acts centrally to stimulate α-MSH production, which, through activation of melanocortin receptors and downstream cAMP-PKA- cAMP Response Element-Binding protein (CREB) signaling, further enhances D2 expression in muscle. Through these converging pathways, leptin increases local T3 production in SkM, thereby amplifying TH-dependent metabolic processes.

This adipose-muscle-thyroid axis has important implications for whole-body energy metabolism. By promoting intracellular TH activation, leptin enhances glucose uptake, fatty acid oxidation, and mitochondrial activity in SkM, contributing to increased energy expenditure. In humans, this mechanism may act as a compensatory response to positive energy balance: increased adiposity elevates leptin levels, which in turn stimulate D2-mediated T3 production and promote a hypermetabolic state aimed at restoring energy equilibrium (Miro et al., 2025b). However, this regulatory axis becomes disrupted in obesity. In leptin-resistant states, despite elevated circulating leptin levels, the ability of leptin to induce D2 expression and enhance intracellular TH activation is impaired. Consistently, D2 expression is increased in moderately overweight individuals but reduced or dysregulated in obese, leptin-resistant subjects. This loss of leptin-dependent TH activation may contribute to reduced metabolic flexibility, decreased energy expenditure, and further weight gain. Supporting this concept, experimental models of impaired TH signaling fail to mount the expected increase in energy expenditure in response to leptin, indicating that TH activation is required for the full metabolic effects of leptin (Miro et al., 2025b).

Collectively, these findings position THs and leptin within an integrated regulatory network that links adipose tissue, central appetite control, and peripheral energy metabolism. Through the modulation of both systemic TH production and tissue-specific TH activation, leptin acts as a key upstream signal that couples energy stores to metabolic output (**Figure 3**). Modulation of this crosstalk, particularly at the level of intracellular TH activation, may represent a critical step in the development of obesity and metabolic syndrome, and highlights potential targets for therapeutic intervention aimed at restoring metabolic homeostasis.

## 5 Circadian organization of the hypothalamic-pituitary-thyroid axis

**Figure 4:**
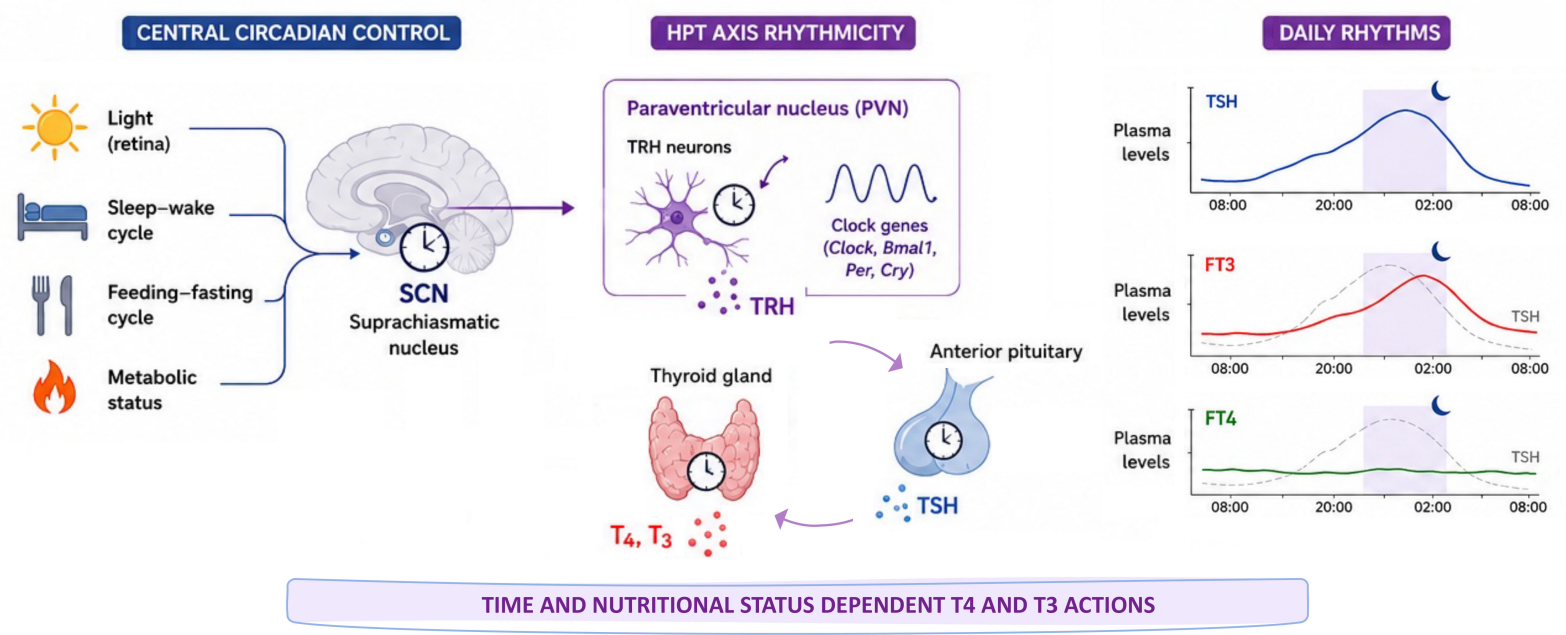
Circadian rhythms and nutritional cues influence thyroid hormone action. The figure depicts the environmental and metabolic inputs, including light exposure (via retinal stimulation), the sleep-wake cycle, feeding-fasting patterns, and Basal Metabolic Rate (BMR), at the level of the suprachiasmatic nucleus (SCN), the central circadian pacemaker. SCN-mediated signaling coordinates hypothalamic control of the Hypothalamus-Pituitary-Thyroid (HPT) axis, leading to rhythmic modulation of its feedback regulation. This results in diurnal fluctuations in circulating Thyroid-Stimulating Hormone (TSH) and Thyroid Hormones (THs), free triiodothyronine (fT3) and free thyroxine (FT4).

Beyond the increased energy demand associated with exercise and the excess energy storage characteristic of obesity, a third level of TH signaling regulation is represented by the sensitivity of the HPT axis to circadian organization and rhythmic cues. The HPT axis is tightly embedded within the circadian timing system, ensuring temporal coordination between endocrine outputs and organismal metabolic demands (**Figure 4**). At the apex of this system, the suprachiasmatic nucleus (SCN) integrates photic input and synchronizes downstream neuroendocrine pathways, including TRH neurons in the paraventricular nucleus (PVN). This hierarchical organization enables the generation of daily oscillations in TSH secretion and, consequently, TH production. Accordingly, the circadian rhythm of TH release is primarily driven by SCN-controlled TSH secretion rather than by an autonomous thyroid clock (Ikegami et al., 2019). However, circadian regulation of the HPT axis reflects a complex interplay between central signals, behavioral cues (sleep-wake cycles, feeding/fasting), and metabolic status (Kalsbeek et al., 2008).

At the molecular level, circadian regulation of the HPT axis is mediated by canonical clock genes (e.g., *Clock*, *Bmal1*, *Per*, *Cry*), which generate ~24-hour oscillations influencing endocrine outputs through transcriptional and neuronal mechanisms (Miro et al., 2023a). Rhythmic expression of clock genes has been demonstrated in TRH neurons and in tanycytes lining the third ventricle, key regulators of local TH metabolism, also display clock gene expression, positioning them as critical mediators linking central circadian signals to TH availability within the hypothalamus (Alkemade et al., 2005).

In addition, TH themselves can modulate clock gene expression and circadian phase in peripheral tissues, suggesting a feedback loop whereby the HPT axis both receives and shapes circadian information. This reciprocal interaction may be particularly relevant under conditions of metabolic stress or endocrine disruption, where altered thyroid status can lead to desynchronization of peripheral clocks and contribute to metabolic dysfunction (Rijo-Ferreira and Takahashi, 2019).

### 5.1 Daily rhythms of TSH secretion

TSH secretion exhibits a robust circadian pattern among pituitary hormones. Neural projections from the SCN, relayed via the subparaventricular zone and dorsomedial hypothalamus, to hypophysiotropic TRH neurons provide the anatomical basis for the circadian regulation of TRH synthesis and secretion (Covarrubias et al., 1994; Kalsbeek et al., 2000; Fliers et al., 2014). Circulating TSH levels begin to rise in the late afternoon, peak during the early part of the night (approximately 02:00-04:00), and decline sharply toward a daytime nadir. This rhythm persists under constant routine conditions, indicating that it is endogenously generated rather than solely driven by external cues. However, its amplitude and phase are strongly modulated by sleep, with sleep onset exerting an inhibitory effect on TSH secretion, thereby shaping the nocturnal profile (Spiegel et al., 1999; Russell et al., 2008) (**Figure 4**).

Mechanistically, SCN output reaches TRH neurons in the PVN via multisynaptic pathways involving the dorsomedial hypothalamus and autonomic nervous system components. Neurotransmitters such as glutamate and gamma-aminobutyric acid (GABA), as well as neuropeptides such as vasopressin, contribute to this regulation. Notably, vasopressinergic projections from the SCN have been implicated in conveying circadian timing signals that influence PVN activity (Kalsbeek et al., 2008). In parallel, ultradian pulsatility of TSH secretion is superimposed upon its circadian rhythm, reflecting pituitary-level regulatory mechanisms and feedback loops.

The role of sleep in modulating TSH dynamics is particularly relevant. Experimental sleep deprivation leads to a marked suppression of the nocturnal TSH surge, while recovery sleep restores its amplitude, indicating that sleep exerts a permissive or gating function. Furthermore, circadian misalignment, such as that observed in shift workers or jet lag, results in phase shifts and attenuation of TSH rhythms, which may have downstream metabolic consequences (Shekhar et al., 2021).

From a clinical perspective, alterations in TSH rhythmicity have been reported in aging, where the amplitude of the nocturnal surge is reduced, and in pathological states such as central hypothyroidism, Non-Thyroidal Illness Syndrome (NTIS), and critical illness. In NTIS, for instance, TSH rhythmicity is often blunted or absent, reflecting disruption of hypothalamic-pituitary regulation. These observations underscore the importance of temporal context in interpreting TSH measurements and suggest that single-time-point assessments may not fully capture thyroid axis function (Fliers et al., 2014).

### 5.2 Circadian fluctuations in T3 and T4 levels

Compared to TSH, circulating levels of T3 and T4 display relatively modest circadian variation, primarily due to their longer half-lives and large distribution volumes. Nevertheless, measurable daily oscillations have been documented, particularly for free T3 (fT3), which exhibits a clear but low-amplitude circadian rhythm. Detailed time-series analyses have shown that fT3 levels follow a sinusoidal pattern that closely parallels TSH, albeit with a consistent phase delay of approximately 1–2 hours, supporting the notion that its rhythmicity is largely driven by TSH-dependent thyroidal secretion and/or peripheral conversion processes. Accordingly, fT3 concentrations typically peak in the early morning hours, shortly after the nocturnal TSH surge, and decline during the afternoon (**Figure 4**).

Overall, these findings indicate that, among circulating THs, fT3 retains the most discernible circadian signature and may represent the most sensitive peripheral readout of central HPT axis rhythmicity (Russell et al., 2008).

The modest but measurable circadian oscillations of circulating T3 and T4 cannot be fully explained by TSH dynamics alone, highlighting the importance of peripheral regulatory mechanisms mediated by Deiodinases.

Accumulating evidence from animal models indicates that the expression and activity of these enzymes exhibit circadian oscillations, driven by both intrinsic molecular clocks and external cues such as feeding time and energy status. For example, D2 expression in the hypothalamus and BAT displays robust rhythmicity, contributing to time-of-day-dependent fluctuations in intracellular T3 concentrations and, consequently, to the temporal regulation of thermogenesis and energy expenditure.

In addition to deiodination, circadian variation in TH transporters, such as MCT8 and OATP1C1, as well as in TH-binding proteins, may further influence hormone distribution and bioavailability. These transporters exhibit a specific expression patterns and, in some cases, rhythmic regulation, thereby contributing to time-of-day-dependent differences in cellular TH uptake, particularly in the brain and other metabolically active tissues (Muller and Heuer, 2014) (Visser et al., 2011). In parallel, fluctuations in circulating binding proteins, including Thyroxine-Binding Globulin (TBG), Transthyretin (TTR), and albumin, may subtly modulate the free fraction of THs, adding a layer of temporal regulation. Together, these mechanisms can lead to a partial dissociation between serum hormone levels and intracellular TH signaling, emphasizing that circulating T3 and T4 concentrations do not necessarily reflect local thyroid status across the day (Bianco et al., 2019).

Another important consideration is the impact of nutritional status and feeding rhythms. Time-restricted feeding and fasting have been shown to markedly influence peripheral TH metabolism, particularly through modulation of Deiodinase expression and activity (**Figure 4**). For instance, alterations in feeding time can shift the phase of D2 rhythmicity in liver and BAT, leading to corresponding changes in local T3 availability independently of TSH fluctuations (Bianco et al., 2019). Fasting, in contrast, is associated with reduced D1 and D2 activity and increased D3 expression in specific tissues, contributing to a local hypothyroid state despite relatively stable circulating hormone levels. These findings indicate that metabolic cues can override or modulate central circadian control of the HPT axis, providing a mechanism for adaptive, intra-tissue regulation of TH action in response to energy availability and environmental conditions (Fliers et al., 2014).

### 5.3 Peripheral clocks and tissue-specific rhythmicity

Peripheral clocks are key determinants of TH action, conferring temporal specificity at the tissue level. Although the HPT axis tightly regulates TH synthesis and secretion, circulating TH levels exhibit low-amplitude or absent circadian oscillations (Van Cauter et al., 1991; Russell et al., 2008; Bautista et al., 2025). This indicates that temporal regulation of TH action is largely governed by peripheral mechanisms downstream of systemic endocrine signals. Importantly, TH-dependent transcriptional responses are modulated in a time-of-day-dependent manner. For example, hepatocyte sensitivity to T3 is temporally gated, suggesting that circadian clocks define permissive windows for hormone action rather than being directly driven by TH rhythms (Lincoln et al., 2024).

Experimental manipulation of thyroid status further supports this model. Hyperthyroidism increases energy expenditure and body temperature and reshapes the amplitude, phase, and Mid-line Estimating Statistic Of Rhythm (MESOR) of metabolic genes, particularly in the liver (Mullur et al., 2014; Sinha et al., 2018; de Assis et al., 2022). In contrast, hypothyroidism has milder effects on the circadian transcriptome (de Assis et al., 2024). Notably, core clock gene expression remains largely preserved across thyroid states, indicating that TH act predominantly downstream of the molecular clock (de Assis et al., 2022; de Assis et al., 2024). These findings support the concept of TH as “circadian tuners” (or tongeber) rather than classical zeitgebers (Bautista et al., 2025). Rather than entraining circadian clocks, THs act as relatively tonic signals that modulate the amplitude and phase of downstream rhythmic processes. This modulation likely involves rhythmic TH transport, local metabolism by Deiodinases, and oscillations in receptor activity, generating time-of-day-dependent responsiveness (Bautista et al., 2025).

This layered regulation is particularly evident in metabolic tissues. In the liver, circadian oscillations in TH receptor activity and Deiodinase expression contribute to daily rhythms in lipid metabolism, gluconeogenesis, and cholesterol homeostasis. Disruption of hepatic clock genes impairs TH-mediated transcriptional responses, indicating that intact circadian machinery is required for optimal hormone action (Yamamoto et al., 2004). Similarly, in SkM, TH regulate mitochondrial function and energy expenditure in a time-dependent manner, influencing metabolic flexibility. BAT exemplifies the integration between circadian clocks and TH signaling: local D2-mediated T3 production is rhythmically regulated and aligned with periods of increased thermogenic demand. In WAT, circadian control of TH signaling influences lipolysis and adipokine secretion, linking the HPT axis to systemic metabolic homeostasis (Bianco et al., 2019). At the central level, tanycytes play a key role in circadian regulation of hypothalamic TH availability. These glial cells control local T3 production via D2 and D3 and exhibit circadian gene expression, acting as interfaces between circulating signals and neuronal circuits regulating energy balance (Alkemade et al., 2005).

Disruption of circadian rhythms, due to genetic, environmental, or behavioral factors, impairs TH signaling and promotes metabolic dysregulation. Conversely, altered thyroid states can feedback on peripheral clocks, inducing phase shifts and reduced amplitude of circadian gene expression. This bidirectional relationship identifies the HPT axis as both a target and a modulator of circadian timing. Overall, the HPT axis operates as a multi-layered circadian system integrating central and peripheral clocks with endocrine and metabolic cues. Despite weak circulating rhythmicity, THs shape metabolic rhythms by tuning cellular responsiveness to intrinsic clock signals. Whether these mechanisms are conserved across organs remains unclear, as evidence outside the liver is still limited (Van Cauter et al., 1991; Russell et al., 2008; de Assis et al., 2022; Lincoln et al., 2024; Bautista et al., 2025; Wang et al., 2026). Elucidating tissue-specific temporal regulation of TH action may have important clinical implications, particularly for metabolic disorders such as metabolic dysfunction-associated steatohepatitis (MASH), where selective TH receptor agonists are emerging as promising therapies.

## 6 Clinical implications and future directions

The emerging view of TH biology as a differentially and dynamically regulated signaling network has important clinical implications. Collectively, the evidence discussed throughout this review supports the concept that circulating TH levels and serum TSH concentrations do not necessarily reflect intracellular TH signaling in different tissues. We propose an “Intracellular TH Signalling Framework”, by which tissues dynamically calibrate TH action in response to metabolic demand, nutritional state, physical activity, and circadian timing (Bianco et al., 2019; Nappi et al., 2025). This organization may explain why individuals with apparently normal circulating thyroid parameters can nevertheless exhibit peripheral alterations in metabolic function. This perspective has several translational implications (Bianco et al., 2019; Russo et al., 2021).

First, it highlights the limitations of relying exclusively on serum TSH as a surrogate marker of tissue euthyroidism. Although TSH remains the cornerstone of thyroid function assessment, variability in transporter expression, Deiodinase activity, receptor isoform distribution, and intracellular T3 buffering may contribute to heterogeneous metabolic phenotypes despite similar serum TH profiles (Groeneweg et al., 2020; Russo et al., 2021). Second, this framework may help explain the persistent symptoms reported by a subset of hypothyroid patients treated with levothyroxine (LT4) monotherapy despite normalization of serum TSH. In this context, genetic variability, including polymorphisms in Deiodinase genes such as Thr92Ala-DIO2, could contribute to interindividual differences in treatment response (Russo et al., 2021). These observations support the need for a more personalized approach to TH replacement based on tissue-specific determinants of TH action. Third, increasing evidence indicates that TH signaling is strongly influenced by circadian and behavioral cues, suggesting that timing may represent an underappreciated determinant of therapeutic efficacy (Bianco et al., 2019). Consequently, chronobiological approaches to TH administration may deserve further investigation, particularly in patients with circadian disruption, shift work, metabolic disease, or altered sleep patterns.

Another major implication concerns the development of tissue-selective therapeutic strategies targeting intracellular TH signaling rather than systemic hormone levels alone. In particular, selective modulation of Deiodinases, TH transporters, or receptor isoforms may offer opportunities to enhance metabolic function in specific organs without globally increasing TH exposure. The development of TRβ-selective agonists has provided proof-of-concept that local TH signaling can be pharmacologically exploited for metabolic disease. Liver-directed thyromimetics, including eprotirome (KB2115), VK2809, and resmetirom (MGL-3196), have demonstrated the ability to improve dyslipidemia, hepatic steatosis, and metabolic dysfunction-associated steatotic liver disease (MASLD/MASH) by enhancing hepatic fatty acid oxidation and cholesterol clearance while limiting cardiac side effects typically associated with TRα activation. Resmetirom has recently emerged as one of the first successful examples of selective TH pathway targeting for MASH treatment, supporting the translational relevance of tissue-specific TH modulation (Ritter et al., 2020; Sinha and Yen, 2024). Additional approaches under investigation include TH metabolites and transporter-targeted therapies. T2 has shown beneficial metabolic effects in experimental models of hepatic steatosis and obesity, whereas thyronamines such as 3-iodothyronamine (T1AM) have demonstrated anti-obesity and metabolic remodeling effects through mechanisms that may be partially independent of classical nuclear TH receptors. Furthermore, TH analogs such as Triac have shown therapeutic potential in disorders associated with impaired TH transport, including MCT8 deficiency, emphasizing the clinical importance of intracellular hormone access in determining tissue TH status (van Geest et al., 2021; Sinha and Yen, 2024).

Collectively, these observations support the concept that future pharmacological strategies may increasingly focus on manipulating intracellular TH signaling in a tissue-restricted manner to improve energy expenditure, mitochondrial function, lipid metabolism, and metabolic flexibility while reducing systemic adverse effects (Sinha et al., 2018).

## 7 Conclusions and perspectives

While traditionally viewed as systemic determinants of BMR, it is now evident that TH action is highly context-dependent and largely shaped by tissue-specific mechanisms controlling intracellular hormone availability and signaling. The integration of hormone transport, local activation and inactivation by Deiodinases, and receptor-mediated transcription defines a multilayered regulatory network that enables precise spatiotemporal control of TH action.

Within this framework, THs emerge as key modulators of metabolic flexibility, allowing organisms to dynamically adapt energy expenditure and substrate utilization to changing physiological conditions. This is particularly evident in contexts such as exercise, where local T3 production supports mitochondrial adaptation and muscle remodeling; in obesity, where crosstalk between TH signaling and adipose-derived factors such as leptin contributes to the regulation of energy balance; and in circadian physiology, where tissue-specific oscillations in TH signaling fine-tune metabolic processes across the day.

A recurring theme throughout this review is the dissociation between circulating TH levels and tissue-specific hormone action. Local control mechanisms, including Deiodinase activity, intracellular hormone buffering, and transporter expression, can generate marked differences in intracellular T3 availability across tissues and physiological states. This highlights a fundamental limitation of relying solely on serum TH measurements to infer metabolic status and underscores the importance of developing approaches that better capture tissue-level TH signaling.

Despite significant advances, several key questions remain unresolved. The relative contribution of circulating versus locally generated T3 to metabolic regulation is still incompletely understood, as is the role of intracellular T3-binding proteins in shaping hormone bioavailability and signaling dynamics. Mechanistically, the discrepancy between circulating and intracellular TH levels may result from alterations in Deiodinases, TH transporters or receptors that impair tissue-specific T3 action despite apparently normal serum T3 concentrations. Experimental evidence from both animal models and human studies supports this concept, showing that defects in TH transporters or receptors can disrupt intracellular T3 availability and downstream signaling (Lopez-Espindola et al., 2014; Bernal et al., 2015; Groeneweg et al., 2020). These mechanisms may help explain why some treated hypothyroid patients continue to experience residual symptoms despite biochemical restoration of TH homeostasis. However, the extent to which these mechanistic findings translate into clinically meaningful outcomes in humans remains unclear, and further studies are needed to clarify the contribution of circulating versus local T3 availability to metabolic homeostasis.

Moreover, the extent to which temporal regulation of TH action, through circadian and metabolic cues, contributes to disease pathogenesis requires further investigation. Addressing these issues will be essential to refine our understanding of TH biology in both physiological and pathological contexts.

From a translational perspective, the tissue-specific nature of TH signaling offers important therapeutic opportunities. Selective targeting of TH pathways, including modulation of Deiodinase activity or the use of receptor isoform-specific agonists, holds promise for the treatment of metabolic disorders such as obesity and MASH. However, the development of such strategies remains challenging, in part due to the complexity and redundancy of TH regulatory mechanisms.

In conclusion, THs should be viewed not simply as systemic metabolic regulators but as dynamic modulators of tissue-specific energy metabolism. A deeper understanding of the mechanisms governing intracellular TH signaling and its temporal organization will be critical for translating basic insights into effective therapeutic strategies for metabolic disease.

## References

[B1] Acampora L. Miro C. Cicatiello A. G. Dentice M. Nappi A. (2025). Deiodinases' inhibitors: A double-edged sword. Front. Biosci. (Landmark Ed) 30, 40246. doi: 10.31083/fbl40246 41074432

[B2] Acampora L. Restolfer F. De Pierro P. Masulli M. Dentice M. Sarnelli G. . (2026). The gut-thyroid axis: physiological regulation of barrier function, microbiota, endocrine signaling and the consequences on energy metabolism. Front. Physiol. 17, 1753136. doi: 10.3389/fphys.2026.1753136 41889794 PMC13012955

[B3] Ahima R. S. Flier J. S. (2000). Leptin. Annu. Rev. Physiol. 62, 413–437. doi: 10.1159/000061014 10845097

[B4] Alkemade A. Friesema E. C. Unmehopa U. A. Fabriek B. O. Kuiper G. G. Leonard J. L. . (2005). Neuroanatomical pathways for thyroid hormone feedback in the human hypothalamus. J. Clin. Endocrinol. Metab. 90, 4322–4334. doi: 10.1210/jc.2004-2567 15840737

[B5] Ambrosio R. Damiano V. Sibilio A. De Stefano M. A. Avvedimento V. E. Salvatore D. . (2013). Epigenetic control of type 2 and 3 deiodinases in myogenesis: role of lysine-specific demethylase enzyme and FoxO3. Nucleic Acids Res. 41, 3551–3562. doi: 10.1093/nar/gkt065 23396445 PMC3616708

[B6] Aranda A. (2021). MicroRNAs and thyroid hormone action. Mol. Cell. Endocrinol. 525, 111175. doi: 10.1016/j.mce.2021.111175 33515639

[B7] Aw D. K. Sinha R. A. Tan H. C. Loh L. M. Salvatore D. Yen P. M. (2014). Studies of molecular mechanisms associated with increased deiodinase 3 expression in a case of consumptive hypothyroidism. J. Clin. Endocrinol. Metab. 99, 3965–3971. doi: 10.1210/jc.2013-3408 24646062

[B8] Barbe P. Larrouy D. Boulanger C. Chevillotte E. Viguerie N. Thalamas C. . (2001). Triiodothyronine-mediated up-regulation of UCP2 and UCP3 mRNA expression in human skeletal muscle without coordinated induction of mitochondrial respiratory chain genes. FASEB J. 15, 13–15. doi: 10.1096/fj.00-0502fje 11099489

[B9] Bassett J. H. Harvey C. B. Williams G. R. (2003). Mechanisms of thyroid hormone receptor-specific nuclear and extra nuclear actions. Mol. Cell. Endocrinol. 213, 1–11. doi: 10.1016/j.mce.2003.10.033 15062569

[B10] Bautista J. Ojeda-Mosquera S. Ordonez-Lozada D. Lopez-Cortes A. (2025). Peripheral clocks and systemic zeitgeber interactions: from molecular mechanisms to circadian precision medicine. Front. Endocrinol. (Lausanne). 16, 1606242. doi: 10.3389/fendo.2025.1606242 40510487 PMC12158691

[B11] Bernal J. Guadano-Ferraz A. Morte B. (2015). Thyroid hormone transporters-functions and clinical implications. Nat. Rev. Endocrinol. 11, 690. doi: 10.1038/nrendo.2015.66 26485690

[B12] Bianco A. C. (2013). Cracking the code for thyroid hormone signaling. Trans. Am. Clin. Climatol. Assoc. 124, 26–35. doi: 10.1210/en.2011-1104 23874007 PMC3715916

[B13] Bianco A. C. Carvalho S. D. Carvalho C. R. Rabelo R. Moriscot A. S. (1998). Thyroxine 5'-deiodination mediates norepinephrine-induced lipogenesis in dispersed brown adipocytes. Endocrinology 139, 571–578. doi: 10.1210/endo.139.2.5737 9449627

[B14] Bianco A. C. Dumitrescu A. Gereben B. Ribeiro M. O. Fonseca T. L. Fernandes G. W. . (2019). Paradigms of dynamic control of thyroid hormone signaling. Endocr. Rev. 40, 1000–1047. doi: 10.1210/er.2018-00275 31033998 PMC6596318

[B15] Bianco A. C. Kieffer J. D. Silva J. E. (1992). Adenosine 3',5'-monophosphate and thyroid hormone control of uncoupling protein messenger ribonucleic acid in freshly dispersed brown adipocytes. Endocrinology 130, 2625–2631. doi: 10.1210/endo.130.5.1374009 1374009

[B16] Bianco A. C. Kim B. S. (2018). Pathophysiological relevance of deiodinase polymorphism. Curr. Opin. Endocrinol. Diabetes Obes. 25, 341–346. doi: 10.1097/med.0000000000000428 30063552 PMC6571023

[B17] Bianco A. C. Silva J. E. (1987). Intracellular conversion of thyroxine to triiodothyronine is required for the optimal thermogenic function of brown adipose tissue. J. Clin. Invest. 79, 295–300. doi: 10.1172/jci112798 3793928 PMC424048

[B18] Bianco A. C. Silva J. E. (1988). Cold exposure rapidly induces virtual saturation of brown adipose tissue nuclear T3 receptors. Am. J. Physiol. 255, E496–E503. doi: 10.1152/ajpendo.1988.255.4.e496 3177636

[B19] Bocco B. M. Louzada R. A. Silvestre D. H. Santos M. C. Anne-Palmer E. Rangel I. F. . (2016). Thyroid hormone activation by type 2 deiodinase mediates exercise-induced peroxisome proliferator-activated receptor-gamma coactivator-1alpha expression in skeletal muscle. J. Physiol. 594, 5255–5269. doi: 10.1113/jp272440 27302464 PMC5023700

[B20] Bochukova E. Schoenmakers N. Agostini M. Schoenmakers E. Rajanayagam O. Keogh J. M. . (2012). A mutation in the thyroid hormone receptor alpha gene. N. Engl. J. Med. 366, 243–249. doi: 10.1056/nejmoa1110296 22168587

[B21] Branco M. Ribeiro M. Negrao N. Bianco A. C. (1999). 3,5,3'-Triiodothyronine actively stimulates UCP in brown fat under minimal sympathetic activity. Am. J. Physiol. 276, E179–E187. doi: 10.1152/ajpendo.1999.276.1.e179 9886965

[B22] Campos-Barros A. Amma L. L. Faris J. S. Shailam R. Kelley M. W. Forrest D. (2000). Type 2 iodothyronine deiodinase expression in the cochlea before the onset of hearing. Proc. Natl. Acad. Sci. U.S.A. 97, 1287–1292. doi: 10.1073/pnas.97.3.1287 10655523 PMC15599

[B23] Canani L. H. Capp C. Dora J. M. Meyer E. L. Wagner M. S. Harney J. W. . (2005). The type 2 deiodinase A/G (Thr92Ala) polymorphism is associated with decreased enzyme velocity and increased insulin resistance in patients with type 2 diabetes mellitus. J. Clin. Endocrinol. Metab. 90, 3472–3478. doi: 10.1210/jc.2004-1977 15797963

[B24] Canto C. Gerhart-Hines Z. Feige J. N. Lagouge M. Noriega L. Milne J. C. . (2009). AMPK regulates energy expenditure by modulating NAD+ metabolism and SIRT1 activity. Nature 458, 1056–1060. doi: 10.1038/nature07813 19262508 PMC3616311

[B25] Carvalho S. D. Kimura E. T. Bianco A. C. Silva J. E. (1991). Central role of brown adipose tissue thyroxine 5'-deiodinase on thyroid hormone-dependent thermogenic response to cold. Endocrinology 128, 2149–2159. doi: 10.1210/endo-128-4-2149 2004619

[B26] Castagna M. G. Dentice M. Cantara S. Ambrosio R. Maino F. Porcelli T. . (2017). DIO2 Thr92Ala reduces deiodinase-2 activity and serum-T3 levels in thyroid-deficient patients. J. Clin. Endocrinol. Metab. 102, 1623–1630. doi: 10.1210/jc.2016-2587 28324063

[B27] Chan J. L. Heist K. DePaoli A. M. Veldhuis J. D. Mantzoros C. S. (2003). The role of falling leptin levels in the neuroendocrine and metabolic adaptation to short-term starvation in healthy men. J. Clin. Invest. 111, 1409–1421. doi: 10.1172/jci200317490 12727933 PMC154448

[B28] Chaves C. Bruinstroop E. Refetoff S. Yen P. M. Anselmo J. (2021). Increased hepatic fat content in patients with resistance to thyroid hormone beta. Thyroid 31, 1127–1134. doi: 10.1089/thy.2020.0651 33353459 PMC8290309

[B29] Chen H. Charlat O. Tartaglia L. A. Woolf E. A. Weng X. Ellis S. J. . (1996). Evidence that the diabetes gene encodes the leptin receptor: identification of a mutation in the leptin receptor gene in db/db mice. Cell. 84, 491–495. doi: 10.1016/s0092-8674(00)81294-5 8608603

[B30] Chua S. C. Chung W. K. Wu-Peng X. S. Zhang Y. Liu S. M. Tartaglia L. . (1996). Phenotypes of mouse diabetes and rat fatty due to mutations in the OB (leptin) receptor. Science 271, 994–996. doi: 10.1126/science.271.5251.994 8584938

[B31] Cicatiello A. G. Di Girolamo D. Dentice M. (2018). Metabolic effects of the intracellular regulation of thyroid hormone: Old players, new concepts. Front. Endocrinol. (Lausanne) 9, 474. doi: 10.3389/fendo.2018.00474 30254607 PMC6141630

[B32] Cicatiello A. G. Iula D. V. Pagliuca C. Pastore G. Pagliarulo C. Catania M. R. . (2014). Identification of Inquilinus limosus in cystic fibrosis: a first report in Italy. New Microbiol. 37, 567–571. 25387296

[B33] Cicatiello A. G. Nappi A. Franchini F. Nettore I. C. Raia M. Rocca C. . (2024). The histone methyltransferase SMYD1 is induced by thermogenic stimuli in adipose tissue. Epigenomics 16, 359–374. doi: 10.2217/epi-2023-0381 38440863

[B34] Cicatiello A. G. Sagliocchi S. Nappi A. Di Cicco E. Miro C. Murolo M. . (2022). Thyroid hormone regulates glutamine metabolism and anaplerotic fluxes by inducing mitochondrial glutamate aminotransferase GPT2. Cell Rep. 38, 110562. doi: 10.1016/j.celrep.2022.110562 35320718 PMC8961412

[B35] Colicchio R. Pagliuca C. Pastore G. Cicatiello A. G. Pagliarulo C. Tala A. . (2015). Fitness cost of rifampin resistance in Neisseria meningitidis: *In vitro* study of mechanisms associated with rpoB H553Y mutation. Antimicrob. Agents Chemother. 59, 7637–7649. doi: 10.1128/aac.01746-15 26416867 PMC4649176

[B36] Considine R. V. Sinha M. K. Heiman M. L. Kriauciunas A. Stephens T. W. Nyce M. R. . (1996). Serum immunoreactive-leptin concentrations in normal-weight and obese humans. N. Engl. J. Med. 334, 292–295. doi: 10.1056/nejm199602013340503 8532024

[B37] Coppola A. Hughes J. Esposito E. Schiavo L. Meli R. Diano S. (2005). Suppression of hypothalamic deiodinase type II activity blunts TRH mRNA decline during fasting. FEBS Lett. 579, 4654–4658. doi: 10.1016/j.febslet.2005.07.035 16098513

[B38] Covarrubias L. Redondo J. L. Vargas M. A. Uribe R. M. Mendez M. Joseph-Bravo P. . (1994). *In vitro* TRH release from hypothalamus slices varies during the diurnal cycle. Neurochem. Res. 19, 845–850. doi: 10.1007/bf00967454 7969755

[B39] Cubuk C. Markowsky H. Herwig A. (2017). Hypothalamic control systems show differential gene expression during spontaneous daily torpor and fasting-induced torpor in the Djungarian hamster (Phodopus sungorus). PloS One 12, e0186299. doi: 10.1371/journal.pone.0186299 29023516 PMC5638525

[B40] da-Silva W. S. Harney J. W. Kim B. W. Li J. Bianco S. D. Crescenzi A. . (2007). The small polyphenolic molecule kaempferol increases cellular energy expenditure and thyroid hormone activation. Diabetes 56, 767–776. doi: 10.2337/db06-1488 17327447

[B41] Davis P. J. Leonard J. L. Lin H. Y. Leinung M. Mousa S. A. (2018). Molecular basis of nongenomic actions of thyroid hormone. Vitam Horm. 106, 67–96. doi: 10.1016/bs.vh.2017.06.001 29407448

[B42] Davis P. J. Mousa S. A. Lin H. Y. (2021). Nongenomic actions of thyroid hormone: the integrin component. Physiol. Rev. 101, 319–352. doi: 10.1152/physrev.00038.2019 32584192

[B43] de Assis L. V. M. Harder L. Lacerda J. T. Parsons R. Kaehler M. Cascorbi I. . (2022). Rewiring of liver diurnal transcriptome rhythms by triiodothyronine (T(3)) supplementation. Elife. 11, 640. doi: 10.1530/endoabs.84.op-04-20 35894384 PMC9391036

[B44] de Assis L. V. M. Harder L. Lacerda J. T. Parsons R. Kaehler M. Cascorbi I. . (2024). Tuning of liver circadian transcriptome rhythms by thyroid hormone state in male mice. Sci. Rep. 14, 640. doi: 10.1038/s41598-023-50374-z 38182610 PMC10770409

[B45] de Lange P. Lanni A. Beneduce L. Moreno M. Lombardi A. Silvestri E. . (2001). Uncoupling protein-3 is a molecular determinant for the regulation of resting metabolic rate by thyroid hormone. Endocrinology 142, 3414–3420. doi: 10.1210/endo.142.8.8303 11459785

[B46] Dentice M. Ambrosio R. Damiano V. Sibilio A. Luongo C. Guardiola O. . (2014). Intracellular inactivation of thyroid hormone is a survival mechanism for muscle stem cell proliferation and lineage progression. Cell Metab. 20, 1038–1048. doi: 10.1016/j.cmet.2014.10.009 25456740 PMC4261081

[B47] Dentice M. Luongo C. Huang S. Ambrosio R. Elefante A. Mirebeau-Prunier D. . (2007). Sonic hedgehog-induced type 3 deiodinase blocks thyroid hormone action enhancing proliferation of normal and Malignant keratinocytes. Proc. Natl. Acad. Sci. U.S.A. 104, 14466–14471. doi: 10.1073/pnas.0706754104 17720805 PMC1964817

[B48] Dentice M. Marsili A. Ambrosio R. Guardiola O. Sibilio A. Paik J. H. . (2010). The FoxO3/type 2 deiodinase pathway is required for normal mouse myogenesis and muscle regeneration. J. Clin. Invest. 120, 4021–4030. doi: 10.1172/jci43670 20978344 PMC2964991

[B49] Dentice M. Marsili A. Zavacki A. Larsen P. R. Salvatore D. (2013). The deiodinases and the control of intracellular thyroid hormone signaling during cellular differentiation. Biochim. Biophys. Acta 1830, 3937–3945. doi: 10.1016/j.bbagen.2012.05.007 22634734 PMC3670672

[B50] Di Cosmo C. Liao X. H. Ye H. Ferrara A. M. Weiss R. E. Refetoff S. . (2013). Mct8-deficient mice have increased energy expenditure and reduced fat mass that is abrogated by normalization of serum T3 levels. Endocrinology 154, 4885–4895. doi: 10.1210/en.2013-1150 24029243 PMC3836073

[B51] Di Girolamo D. Ambrosio R. De Stefano M. A. Mancino G. Porcelli T. Luongo C. . (2016). Reciprocal interplay between thyroid hormone and microRNA-21 regulates hedgehog pathway-driven skin tumorigenesis. J. Clin. Invest. 126, 2308–2320. doi: 10.1172/jci84465 27159391 PMC4887175

[B52] Di Girolamo D. Di Cicco E. Miro C. Murolo M. Nappi A. Cicatiello A. G. (2026). Thyroid hormone inactivation sustains cancer stem cell maintenance and tumorigenesis in basal cell carcinoma. J. Invest. Dermatol. S0022-202X(26)00074-6. doi: 10.1016/j.jid.2026.01.025 41644085

[B53] Dillman W. Surks M. I. Oppenheimer J. H. (1974). Quantitative aspects of iodothyronine binding by cytosol proteins of rat liver and kidney. Endocrinology 95, 492–498. doi: 10.1210/endo-95-2-492 4369099

[B54] Dora J. M. MaChado W. E. Rheinheimer J. Crispim D. Maia A. L. (2010). Association of the type 2 deiodinase Thr92Ala polymorphism with type 2 diabetes: case-control study and meta-analysis. Eur. J. Endocrinol. 163, 427–434. doi: 10.1530/eje-10-0419 20566590

[B55] Dore R. Watson L. Hollidge S. Krause C. Sentis S. C. Oelkrug R. . (2023). Resistance to thyroid hormone induced tachycardia in RTHalpha syndrome. Nat. Commun. 14, 3312. doi: 10.1038/s41467-023-38960-1 37286550 PMC10247713

[B56] Dumitrescu A. M. Di Cosmo C. Liao X. H. Weiss R. E. Refetoff S. (2010). The syndrome of inherited partial SBP2 deficiency in humans. Antioxid. Redox Signal. 12, 905–920. doi: 10.1089/ars.2009.2892 19769464 PMC2864657

[B57] Dumitrescu A. M. Liao X. H. Best T. B. Brockmann K. Refetoff S. (2004). A novel syndrome combining thyroid and neurological abnormalities is associated with mutations in a monocarboxylate transporter gene. Am. J. Hum. Genet. 74, 168–175. doi: 10.1086/380999 14661163 PMC1181904

[B58] Fan W. Boston B. A. Kesterson R. A. Hruby V. J. Cone R. D. (1997). Role of melanocortinergic neurons in feeding and the agouti obesity syndrome. Nature 385, 165–168. doi: 10.1038/385165a0 8990120

[B59] Farooqi I. S. Jebb S. A. Langmack G. Lawrence E. Cheetham C. H. Prentice A. M. . (1999). Effects of recombinant leptin therapy in a child with congenital leptin deficiency. N. Engl. J. Med. 341, 879–884. doi: 10.1056/nejm199909163411204 10486419

[B60] Fliers E. Kalsbeek A. Boelen A. (2014). Beyond the fixed setpoint of the hypothalamus-pituitary-thyroid axis. Eur. J. Endocrinol. 171, R197–R208. doi: 10.1007/978-3-540-29678-2_2325 25005935

[B61] Fonseca T. L. Fernandes G. W. McAninch E. A. Bocco B. M. Abdalla S. M. Ribeiro M. O. . (2015). Perinatal deiodinase 2 expression in hepatocytes defines epigenetic susceptibility to liver steatosis and obesity. Proc. Natl. Acad. Sci. U.S.A. 112, 14018–14023. doi: 10.1073/pnas.1508943112 26508642 PMC4653175

[B62] Friesema E. C. Grueters A. Biebermann H. Krude H. von Moers A. Reeser M. . (2004). Association between mutations in a thyroid hormone transporter and severe X-linked psychomotor retardation. Lancet 364, 1435–1437. doi: 10.1016/s0140-6736(04)17226-7 15488219

[B63] Friesema E. C. Jansen J. Jachtenberg J. W. Visser W. E. Kester M. H. Visser T. J. (2008). Effective cellular uptake and efflux of thyroid hormone by human monocarboxylate transporter 10. Mol. Endocrinol. 22, 1357–1369. doi: 10.1210/me.2007-0112 18337592 PMC5419535

[B64] Fu J. Fujisawa H. Follman B. Liao X. H. Dumitrescu A. M. (2017). Thyroid hormone metabolism defects in a mouse model of SBP2 deficiency. Endocrinology 158, 4317–4330. doi: 10.1210/en.2017-00618 29029094 PMC5711384

[B65] Galton V. A. Hernandez A. (2023). Thyroid hormone metabolism: A historical perspective. Thyroid 33, 24–31. doi: 10.1089/thy.2022.0161 35699066 PMC9885541

[B66] Gereben B. McAninch E. A. Ribeiro M. O. Bianco A. C. (2015). Scope and limitations of iodothyronine deiodinases in hypothyroidism. Nat. Rev. Endocrinol. 11, 642–652. doi: 10.1038/nrendo.2015.155 26416219 PMC5003781

[B67] Gil-Ibanez P. Belinchon M. M. Morte B. Obregon M. J. Bernal J. (2017). Is the intrinsic genomic activity of thyroxine relevant *in vivo*? Effects on gene expression in primary cerebrocortical and neuroblastoma cells. Thyroid 27, 1092–1098. doi: 10.1089/thy.2017.0024 28605984

[B68] Gonzalez O. Gonzalez E. Sanchez C. Pinto J. Gonzalez I. Enriquez O. . (1998). Effect of exercise on erythrocyte beta-adrenergic receptors and plasma concentrations of catecholamines and thyroid hormones in Thoroughbred horses. Equine Vet. J. 30, 72–78. doi: 10.1007/978-3-319-56782-2_1760-2 9458402

[B69] Greenwood B. N. Fleshner M. (2011). Exercise, stress resistance, and central serotonergic systems. Exerc Sport Sci. Rev. 39, 140–149. doi: 10.1097/jes.0b013e31821f7e45 21508844 PMC4303035

[B70] Groeneweg S. van Geest F. S. Peeters R. P. Heuer H. Visser W. E. (2020). Thyroid hormone transporters. Endocr. Rev. 41, 1–55. doi: 10.1210/endrev/bnz008 31754699

[B71] Grontved L. Waterfall J. J. Kim D. W. Baek S. Sung M. H. Zhao L. . (2015). Transcriptional activation by the thyroid hormone receptor through ligand-dependent receptor recruitment and chromatin remodelling. Nat. Commun. 6, 7048. doi: 10.1038/ncomms8048 25916672 PMC6309829

[B72] Grozovsky R. Ribich S. Rosene M. L. Mulcahey M. A. Huang S. A. Patti M. E. . (2009). Type 2 deiodinase expression is induced by peroxisomal proliferator-activated receptor-gamma agonists in skeletal myocytes. Endocrinology 150, 1976–1983. doi: 10.1210/en.2008-0938 19036883 PMC2659265

[B73] Grumati P. Coletto L. Sabatelli P. Cescon M. Angelin A. Bertaggia E. . (2010). Autophagy is defective in collagen VI muscular dystrophies, and its reactivation rescues myofiber degeneration. Nat. Med. 16, 1313–1320. doi: 10.1038/nm.2247 21037586

[B74] Gumieniak O. Perlstein T. S. Williams J. S. Hopkins P. N. Brown N. J. Raby B. A. . (2007). Ala92 type 2 deiodinase allele increases risk for the development of hypertension. Hypertension 49, 461–466. doi: 10.1161/01.hyp.0000256295.72185.fd 17224473

[B75] Hashizume K. Miyamoto T. Ichikawa K. Yamauchi K. Kobayashi M. Sakurai A. . (1989). Purification and characterization of NADPH-dependent cytosolic 3,5,3'-triiodo-L-thyronine binding protein in rat kidney. J. Biol. Chem. 264, 4857–4863. doi: 10.1016/s0021-9258(18)83670-5 2925671

[B76] Hermann R. Lay D. Wahl P. Roth W. T. Petrowski K. (2019). Effects of psychosocial and physical stress on lactate and anxiety levels. Stress 22, 664–669. doi: 10.1080/10253890.2019.1610743 31062999

[B77] Hermann R. Schaller A. Lay D. Bloch W. Albus C. Petrowski K. (2021). Effect of acute psychosocial stress on the brain-derived neurotrophic factor in humans - a randomized cross within trial. Stress 24, 442–449. doi: 10.1080/10253890.2020.1854218 33236949

[B78] Horowitz B. Hensley C. B. Quintero M. Azuma K. K. Putnam D. McDonough A. A. (1990). Differential regulation of Na,K-ATPase alpha 1, alpha 2, and beta subunit mRNA and protein levels by thyroid hormone. J. Biol. Chem. 265, 14308–14314. doi: 10.1016/s0021-9258(18)77301-8 2167313

[B79] Hosoi Y. Murakami M. Mizuma H. Ogiwara T. Imamura M. Mori M. (1999). Expression and regulation of type II iodothyronine deiodinase in cultured human skeletal muscle cells. J. Clin. Endocrinol. Metab. 84, 3293–3300. doi: 10.1210/jc.84.9.3293 10487701

[B80] Ikegami K. Refetoff S. Van Cauter E. Yoshimura T. (2019). Interconnection between circadian clocks and thyroid function. Nat. Rev. Endocrinol. 15, 590–600. doi: 10.1038/s41574-019-0237-z 31406343 PMC7288350

[B81] Jo S. Fonseca T. L. Bocco B. Fernandes G. W. McAninch E. A. Bolin A. P. . (2019). Type 2 deiodinase polymorphism causes ER stress and hypothyroidism in the brain. J. Clin. Invest. 129, 230–245. doi: 10.1172/jci123176 30352046 PMC6307951

[B82] Kalsbeek A. Fliers E. Franke A. N. Wortel J. Buijs R. M. (2000). Functional connections between the suprachiasmatic nucleus and the thyroid gland as revealed by lesioning and viral tracing techniques in the rat. Endocrinology. 141, 3832–3841. doi: 10.1210/endo.141.10.7709 11014240

[B83] Kalsbeek A. Foppen E. Schalij I. Van Heijningen C. van der Vliet J. Fliers E. . (2008). Circadian control of the daily plasma glucose rhythm: an interplay of GABA and glutamate. PloS One 3, e3194. doi: 10.1371/journal.pone.0003194 18791643 PMC2527681

[B84] Kester M. H. Martinez de Mena R. Obregon M. J. Marinkovic D. Howatson A. Visser T. J. . (2004). Iodothyronine levels in the human developing brain: Major regulatory roles of iodothyronine deiodinases in different areas. J. Clin. Endocrinol. Metab. 89, 3117–3128. doi: 10.1210/jc.2003-031832 15240580

[B85] Kim J. D. Leyva S. Diano S. (2014). Hormonal regulation of the hypothalamic melanocortin system. Front. Physiol. 5, 480. doi: 10.3389/fphys.2014.00480 25538630 PMC4260486

[B86] Kimura K. Sasaki N. Asano A. Mizukami J. Kayahashi S. Kawada T. . (2000). Mutated human beta3-adrenergic receptor (Trp64Arg) lowers the response to beta3-adrenergic agonists in transfected 3T3-L1 preadipocytes. Horm. Metab. Res. 32, 91–96. doi: 10.1055/s-2007-978597 10786926

[B87] Kinney C. J. Bloch R. J. (2021). Micro-crystallin: a thyroid hormone binding protein. Endocr. Regul. 55, 89–102. doi: 10.2478/enr-2021-0011 34020530 PMC9202446

[B88] Lartey L. J. Werneck-de-Castro J. P. I O. S. Unterman T. G. Bianco A. C. (2015). Coupling between nutrient availability and thyroid hormone activation. J. Biol. Chem. 290, 30551–30561. doi: 10.1074/jbc.m115.665505 26499800 PMC4683275

[B89] Lee G. H. Proenca R. Montez J. M. Carroll K. M. Darvishzadeh J. G. Lee J. I. . (1996). Abnormal splicing of the leptin receptor in diabetic mice. Nature 379, 632–635. doi: 10.1038/379632a0 8628397

[B90] Legradi G. Emerson C. H. Ahima R. S. Flier J. S. Lechan R. M. (1997). Leptin prevents fasting-induced suppression of prothyrotropin-releasing hormone messenger ribonucleic acid in neurons of the hypothalamic paraventricular nucleus. Endocrinology 138, 2569–2576. doi: 10.1210/endo.138.6.5209 9165050

[B91] Lesmana R. Sinha R. A. Singh B. K. Zhou J. Ohba K. Wu Y. . (2016). Thyroid hormone stimulation of autophagy is essential for mitochondrial biogenesis and activity in skeletal muscle. Endocrinology 157, 23–38. doi: 10.1210/en.2015-1632 26562261

[B92] Liang X. Liu L. Fu T. Zhou Q. Zhou D. Xiao L. . (2016). Exercise inducible lactate dehydrogenase B regulates mitochondrial function in skeletal muscle. J. Biol. Chem. 291, 25306–25318. doi: 10.1074/jbc.m116.749424 27738103 PMC5207234

[B93] Lincoln K. Zhou J. Oster H. de Assis L. V. M. (2024). Circadian gating of thyroid hormone action in hepatocytes. Cells. 13, 1038. doi: 10.3390/cells13121038 38920666 PMC11202020

[B94] Lira V. A. Benton C. R. Yan Z. Bonen A. (2010). PGC-1alpha regulation by exercise training and its influences on muscle function and insulin sensitivity. Am. J. Physiol. Endocrinol. Metab. 299, E145–E161. doi: 10.1152/ajpendo.00755.2009 20371735 PMC2928513

[B95] Liu Y. Y. Brent G. A. (2018). Posttranslational modification of thyroid hormone nuclear receptor by sumoylation. Methods Mol. Biol. 1801, 47–59. doi: 10.1007/978-1-4939-7902-8_6 29892816

[B96] Lombardi A. Senese R. De Matteis R. Busiello R. A. Cioffi F. Goglia F. . (2015). 3,5-Diiodo-L-thyronine activates brown adipose tissue thermogenesis in hypothyroid rats. PloS One 10, e0116498. doi: 10.1371/journal.pone.0116498 25658324 PMC4319745

[B97] Lopez-Espindola D. Morales-Bastos C. Grijota-Martinez C. Liao X. H. Lev D. Sugo E. . (2014). Mutations of the thyroid hormone transporter MCT8 cause prenatal brain damage and persistent hypomyelination. J. Clin. Endocrinol. Metab. 99, E2799–E2804. doi: 10.1210/jc.2014-2162 25222753 PMC4255116

[B98] Lo Verso F. Carnio S. Vainshtein A. Sandri M. (2014). Autophagy is not required to sustain exercise and PRKAA1/AMPK activity but is important to prevent mitochondrial damage during physical activity. Autophagy 10, 1883–1894. doi: 10.4161/auto.32154 25483961 PMC4502666

[B99] Luongo C. Dentice M. Salvatore D. (2019). Deiodinases and their intricate role in thyroid hormone homeostasis. Nat. Rev. Endocrinol. 15, 479–488. doi: 10.1038/s41574-019-0218-2 31160732

[B100] Mastorakos G. Pavlatou M. (2005). Exercise as a stress model and the interplay between the hypothalamus-pituitary-adrenal and the hypothalamus-pituitary-thyroid axes. Horm. Metab. Res. 37, 577–584. doi: 10.1055/s-2005-870426 16175498

[B101] Mayerl S. Muller J. Bauer R. Richert S. Kassmann C. M. Darras V. M. . (2014). Transporters MCT8 and OATP1C1 maintain murine brain thyroid hormone homeostasis. J. Clin. Invest. 124, 1987–1999. doi: 10.1172/jci70324 24691440 PMC4001533

[B102] Mentuccia D. Proietti-Pannunzi L. Tanner K. Bacci V. Pollin T. I. Poehlman E. T. . (2002). Association between a novel variant of the human type 2 deiodinase gene Thr92Ala and insulin resistance: evidence of interaction with the Trp64Arg variant of the beta-3-adrenergic receptor. Diabetes 51, 880–883. doi: 10.2337/diabetes.51.3.880 11872697

[B103] Mikkelsen K. Stojanovska L. Polenakovic M. Bosevski M. Apostolopoulos V. (2017). Exercise and mental health. Maturitas 106, 48–56. doi: 10.1016/j.maturitas.2017.09.003 29150166

[B104] Miro C. Cicatiello A. G. Dentice M. Nappi A. (2025a). The role of deiodinases on metabolic alteration in cancer. Semin. Cancer Biol. 114, 215–226. doi: 10.1016/j.semcancer.2025.07.003 40651548

[B105] Miro C. Cicatiello A. G. Nappi A. Sagliocchi S. Acampora L. Restolfer F. . (2025b). Leptin enhances the intracellular thyroid hormone activation in skeletal muscle to boost energy balance. Cell Metab. 37, 936–953, e7. doi: 10.1016/j.cmet.2026.02.015 39986272

[B106] Miro C. Di Cicco E. Ambrosio R. Mancino G. Di Girolamo D. Cicatiello A. G. . (2019). Thyroid hormone induces progression and invasiveness of squamous cell carcinomas by promoting a ZEB-1/E-cadherin switch. Nat. Commun. 10, 5410. doi: 10.1038/s41467-019-13140-2 31776338 PMC6881453

[B107] Miro C. Docimo A. Barrea L. Verde L. Cernea S. Sojat A. S. . (2023a). Time" for obesity-related cancer: The role of the circadian rhythm in cancer pathogenesis and treatment. Semin. Cancer Biol. 91, 99–109. doi: 10.1016/j.semcancer.2023.03.003 36893964

[B108] Miro C. Nappi A. Sagliocchi S. Di Cicco E. Murolo M. Torabinejad S. . (2023b). Thyroid hormone regulates the lipid content of muscle fibers, thus affecting physical exercise performance. Int. J. Mol. Sci. 24, 12074. doi: 10.3390/ijms241512074 37569453 PMC10418733

[B109] Montague C. T. Farooqi I. S. Whitehead J. P. Soos M. A. Rau H. Wareham N. J. . (1997). Congenital leptin deficiency is associated with severe early-onset obesity in humans. Nature 387, 903–908. doi: 10.1038/43185 9202122

[B110] Moran C. Chatterjee K. (2015). Resistance to thyroid hormone due to defective thyroid receptor alpha. Best Pract. Res. Clin. Endocrinol. Metab. 29, 647–657. doi: 10.1016/j.beem.2015.07.007 26303090 PMC4559105

[B111] Muller J. Heuer H. (2014). Expression pattern of thyroid hormone transporters in the postnatal mouse brain. Front. Endocrinol. (Lausanne) 5, 92. doi: 10.3389/fendo.2014.00092 24994998 PMC4061481

[B112] Mullur R. Liu Y. Y. Brent G. A. (2014). Thyroid hormone regulation of metabolism. Physiol. Rev. 94, 355–382. doi: 10.1152/physrev.00030.2013 24692351 PMC4044302

[B113] Murolo M. Di Vincenzo O. Cicatiello A. G. Scalfi L. Dentice M. (2022). Cardiovascular and neuronal consequences of thyroid hormones alterations in the ischemic stroke. Metabolites 13, 22. doi: 10.3390/metabo13010022 36676947 PMC9863748

[B114] Nappi A. Miro C. Pezone A. Tramontano A. Di Cicco E. Sagliocchi S. . (2023). Loss of p53 activates thyroid hormone via type 2 deiodinase and enhances DNA damage. Nat. Commun. 14, 1244. doi: 10.1038/s41467-023-36755-y 36871014 PMC9985592

[B115] Nappi A. Murolo M. Sagliocchi S. Miro C. Cicatiello A. G. Di Cicco E. . (2021). Selective inhibition of genomic and non-genomic effects of thyroid hormone regulates muscle cell differentiation and metabolic behavior. Int. J. Mol. Sci. 22, 7175. doi: 10.3390/ijms22137175 34281225 PMC8269436

[B116] Nappi A. Sagliocchi S. Cicatiello A. G. Miro C. (2025). Thyroid hormone imbalance, malnutrition, and sarcopenia: A triad of muscle health challenges. J. Basic. Clin. Physiol. Pharmacol. 36, 351–357. doi: 10.1515/jbcpp-2025-0160 41233980

[B117] Nicholson T. Church C. Baker D. J. Jones S. W. (2018). The role of adipokines in skeletal muscle inflammation and insulin sensitivity. J. Inflamm. (Lond) 15, 9. doi: 10.1186/s12950-018-0185-8 29760587 PMC5944154

[B118] Obregon M. J. (2014). Adipose tissues and thyroid hormones. Front. Physiol. 5, 479. doi: 10.3389/fphys.2014.00479 25566082 PMC4263094

[B119] Odriozola A. Gonzalez A. Odriozola I. Corbi F. Alvarez-Herms J. (2025). Thyroid-microbiome allostasis and mitochondrial performance: An integrative perspective in exercise physiology. Nutrients 18, 59. doi: 10.3390/nu18010059 41515177 PMC12787513

[B120] Oppenheimer J. H. Schwartz H. L. (1985). Stereospecific transport of triiodothyronine from plasma to cytosol and from cytosol to nucleus in rat liver, kidney, brain, and heart. J. Clin. Invest. 75, 147–154. doi: 10.1172/jci111667 3965501 PMC423420

[B121] Oshima A. Suzuki S. Takumi Y. Hashizume K. Abe S. Usami S. (2006). CRYM mutations cause deafness through thyroid hormone binding properties in the fibrocytes of the cochlea. J. Med. Genet. 43, e25. doi: 10.1136/jmg.2005.034397 16740909 PMC2564543

[B122] Pagel-Langenickel I. Bao J. Joseph J. J. Schwartz D. R. Mantell B. S. Xu X. . (2008). PGC-1alpha integrates insulin signaling, mitochondrial regulation, and bioenergetic function in skeletal muscle. J. Biol. Chem. 283, 22464–22472. doi: 10.1074/jbc.m800842200 18579525 PMC2504883

[B123] Pagliuca C. Cicatiello A. G. Colicchio R. Greco A. Cerciello R. Auletta L. . (2016). Novel approach for evaluation of Bacteroides fragilis protective role against Bartonella henselae liver damage in immunocompromised murine model. Front. Microbiol. 7, 1750. doi: 10.3389/fmicb.2016.01750 27872616 PMC5097911

[B124] Paolino B. S. Pomerantzeff P. M. Dallan L. A. O. Gaiotto F. A. Preite N. Z. Latronico A. C. . (2017). Myocardial inactivation of thyroid hormones in patients with aortic stenosis. Thyroid 27, 738–745. doi: 10.1089/thy.2016.0514 28095748 PMC5749598

[B125] Pappa T. Ferrara A. M. Refetoff S. (2015). Inherited defects of thyroxine-binding proteins. Best Pract. Res. Clin. Endocrinol. Metab. 29, 735–747. doi: 10.1016/j.beem.2015.09.002 26522458 PMC4632647

[B126] Pelleymounter M. A. Cullen M. J. Baker M. B. Hecht R. Winters D. Boone T. . (1995). Effects of the obese gene product on body weight regulation in ob/ob mice. Science 269, 540–543. doi: 10.1126/science.7624776 7624776

[B127] Petrowski K. Kahaly G. J. (2025). Stress and thyroid function-from bench to bedside. Endocr. Rev. 46, 709–735. doi: 10.1210/endrev/bnaf015 40522816

[B128] Picascia A. Pagliuca C. Sommese L. Colicchio R. Casamassimi A. Labonia F. . (2017). Seroprevalence of Bartonella henselae in patients awaiting heart transplant in Southern Italy. J. Microbiol. Immunol. Infect. 50, 239–244. doi: 10.1016/j.jmii.2015.05.001 26051222

[B129] Ramadan W. Marsili A. Larsen P. R. Zavacki A. M. Silva J. E. (2011). Type-2 iodothyronine 5'deiodinase (D2) in skeletal muscle of C57Bl/6 mice. II. Evidence for a role of D2 in the hypermetabolism of thyroid hormone receptor alpha-deficient mice. Endocrinology 152, 3093–3102. doi: 10.1210/en.2011-0139 21652727 PMC3138235

[B130] Razvi S. Jabbar A. Pingitore A. Danzi S. Biondi B. Klein I. . (2018). Thyroid hormones and cardiovascular function and diseases. J. Am. Coll. Cardiol. 71, 1781–1796. doi: 10.1016/j.jacc.2018.02.045 29673469

[B131] Refetoff S. Weiss R. E. Usala S. J. (1993). The syndromes of resistance to thyroid hormone. Endocr. Rev. 14, 348–399. doi: 10.1152/ajpendo.1982.243.2.e88 8319599

[B132] Rijo-Ferreira F. Takahashi J. S. (2019). Genomics of circadian rhythms in health and disease. Genome Med. 11, 82. doi: 10.1186/s13073-019-0704-0 31847894 PMC6916512

[B133] Ritter M. J. Amano I. Hollenberg A. N. (2020). Thyroid hormone signaling and the liver. Hepatology 72, 742–752. doi: 10.1002/hep.31296 32343421

[B134] Rodd C. Schwartz H. L. Strait K. A. Oppenheimer J. H. (1992). Ontogeny of hepatic nuclear triiodothyronine receptor isoforms in the rat. Endocrinology 131, 2559–2564. doi: 10.1210/en.131.6.2559 1446599

[B135] Russell W. Harrison R. F. Smith N. Darzy K. Shalet S. Weetman A. P. . (2008). Free triiodothyronine has a distinct circadian rhythm that is delayed but parallels thyrotropin levels. J. Clin. Endocrinol. Metab. 93, 2300–2306. doi: 10.1210/jc.2007-2674 18364382

[B136] Russo S. C. Salas-Lucia F. Bianco A. C. (2021). Deiodinases and the metabolic code for thyroid hormone action. Endocrinology 162, bqab059. doi: 10.1210/endocr/bqab059 33720335 PMC8237994

[B137] Sagliocchi S. Cicatiello A. G. Di Cicco E. Ambrosio R. Miro C. Di Girolamo D. . (2019). The thyroid hormone activating enzyme, type 2 deiodinase, induces myogenic differentiation by regulating mitochondrial metabolism and reducing oxidative stress. Redox Biol. 24, 101228. doi: 10.1016/j.redox.2019.101228 31153038 PMC6543119

[B138] Sagliocchi S. Murolo M. Cicatiello A. G. Miro C. Nappi A. Di Cicco E. . (2023). Repositioning of cefuroxime as novel selective inhibitor of the thyroid hormone activating enzyme type 2 deiodinase. Pharmacol. Res. 189, 106685. doi: 10.1530/endoabs.92.ps2-19-04 36773711

[B139] Sagliocchi S. Restolfer F. Cossidente A. Dentice M. (2024). The key roles of thyroid hormone in mitochondrial regulation, at interface of human health and disease. J. Basic. Clin. Physiol. Pharmacol. 35, 231–240. doi: 10.1515/jbcpp-2024-0108 39023546 PMC11522957

[B140] Salvatore D. Simonides W. S. Dentice M. Zavacki A. M. Larsen P. R. (2014). Thyroid hormones and skeletal muscle--new insights and potential implications. Nat. Rev. Endocrinol. 10, 206–214. doi: 10.1038/nrendo.2013.238 24322650 PMC4037849

[B141] Schuler C. B. Sayre A. B. Zakaria L. Tassone S. Rinehart A. Harris R. (2026). Energy allocation resilience and endocrine integration. Int. J. Mol. Sci. 27, 1345. doi: 10.20944/preprints202512.2382.v1 41683768 PMC12898343

[B142] Schweizer U. Steegborn C. (2015). New insights into the structure and mechanism of iodothyronine deiodinases. J. Mol. Endocrinol. 55, R37–R52. doi: 10.1530/jme-15-0156 26390881

[B143] Shekhar S. Hall J. E. Klubo-Gwiezdzinska J. (2021). The hypothalamic pituitary thyroid axis and sleep. Curr. Opin. Endocr. Metab. Res. 17, 8–14. doi: 10.1007/978-981-10-3695-8_22 34322645 PMC8315115

[B144] Simonides W. S. van der Linden G. C. van Hardeveld C. (1990). Thyroid hormone differentially affects mRNA levels of Ca-ATPase isozymes of sarcoplasmic reticulum in fast and slow skeletal muscle. FEBS Lett. 274, 73–76. doi: 10.1016/0014-5793(90)81332-i 2147661

[B145] Simonides W. S. van Hardeveld C. (2008). Thyroid hormone as a determinant of metabolic and contractile phenotype of skeletal muscle. Thyroid 18, 205–216. doi: 10.1089/thy.2007.0256 18279021

[B146] Singh B. K. Sinha R. A. Tripathi M. Mendoza A. Ohba K. Sy J. A. C. . (2018a). Thyroid hormone receptor and ERRalpha coordinately regulate mitochondrial fission, mitophagy, biogenesis, and function. Sci. Signal. 11, eaam5855. doi: 10.1126/scisignal.aam5855 29945885

[B147] Singh B. K. Sinha R. A. Yen P. M. (2018b). Novel transcriptional mechanisms for regulating metabolism by thyroid hormone. Int. J. Mol. Sci. 19, 3284. doi: 10.3390/ijms19103284 30360449 PMC6214012

[B148] Singh B. K. Sinha R. A. Zhou J. Xie S. Y. You S. H. Gauthier K. . (2013). FoxO1 deacetylation regulates thyroid hormone-induced transcription of key hepatic gluconeogenic genes. J. Biol. Chem. 288, 30365–30372. doi: 10.1074/jbc.m113.504845 23995837 PMC3798501

[B149] Sinha R. A. Singh B. K. Yen P. M. (2018). Direct effects of thyroid hormones on hepatic lipid metabolism. Nat. Rev. Endocrinol. 14, 259–269. doi: 10.1038/nrendo.2018.10 29472712 PMC6013028

[B150] Sinha R. A. Yen P. M. (2024). Metabolic messengers: Thyroid hormones. Nat. Metab. 6, 639–650. doi: 10.1038/s42255-024-00986-0 38671149 PMC7615975

[B151] Spiegel K. Leproult R. Van Cauter E. (1999). Impact of sleep debt on metabolic and endocrine function. Lancet. 354, 1435–1439. doi: 10.1016/s0140-6736(99)01376-8 10543671

[B152] Suzuki S. Suzuki N. Mori J. Oshima A. Usami S. Hashizume K. (2007). Micro-crystallin as an intracellular 3,5,3'-triiodothyronine holder *in vivo*. Mol. Endocrinol. 21, 885–894. doi: 10.1210/me.2006-0403 17264173

[B153] Thakran S. Sharma P. Attia R. R. Hori R. T. Deng X. Elam M. B. . (2013). Role of sirtuin 1 in the regulation of hepatic gene expression by thyroid hormone. J. Biol. Chem. 288, 807–818. doi: 10.1074/jbc.m112.437970 23209300 PMC3543030

[B154] Torabinejad S. Miro C. Barone B. Imbimbo C. Crocetto F. Dentice M. (2023). The androgen-thyroid hormone crosstalk in prostate cancer and the clinical implications. Eur. Thyroid J. 12, e220228. doi: 10.1530/etj-22-0228 36930264 PMC10160561

[B155] Triandafillou J. Gwilliam C. Himms-Hagen J. (1982). Role of thyroid hormone in cold-induced changes in rat brown adipose tissue mitochondria. Can. J. Biochem. 60, 530–537. doi: 10.1139/o82-065 7104831

[B156] Uribe R. M. Jaimes-Hoy L. Ramirez-Martinez C. Garcia-Vazquez A. Romero F. Cisneros M. . (2014). Voluntary exercise adapts the hypothalamus-pituitary-thyroid axis in male rats. Endocrinology 155, 2020–2030. doi: 10.1210/en.2013-1724 24605825

[B157] Van Cauter E. Blackman J. D. Roland D. Spire J. P. Refetoff S. Polonsky K. S. (1991). Modulation of glucose regulation and insulin secretion by circadian rhythmicity and sleep. J. Clin. Invest. 88, 934–942. doi: 10.1172/jci115396 1885778 PMC295490

[B158] van Geest F. S. Gunhanlar N. Groeneweg S. Visser W. E. (2021). Monocarboxylate transporter 8 deficiency: from pathophysiological understanding to therapy development. Front. Endocrinol. (Lausanne) 12, 723750. doi: 10.3389/fendo.2021.723750 34539576 PMC8440930

[B159] Verbrugge S. A. J. Gehlert S. Stadhouders L. E. M. Jacko D. Aussieker T. G M. J. D. W. . (2020). PKM2 determines myofiber hypertrophy *in vitro* and increases in response to resistance exercise in human skeletal muscle. Int. J. Mol. Sci. 21, 7062. doi: 10.3390/ijms21197062 32992783 PMC7583908

[B160] Visser W. E. Friesema E. C. Visser T. J. (2011). Minireview: thyroid hormone transporters: the knowns and the unknowns. Mol. Endocrinol. 25, 1–14. doi: 10.1210/edrv.31.4.9986 20660303 PMC5417302

[B161] Wang J. Cao M. Li S. Pei W. Li J. Wang Z. (2026). Circadian clock genes: their influence on liver metabolism, disease development and treatment (review). Mol. Med. Rep. 33, 3. doi: 10.3892/mmr.2025.13713 41104881 PMC12555910

[B162] Wang M. Li Q. Deng A. Zhu X. Yang J. (2020). Identification of a novel mutation in CRYM in a Chinese family with hearing loss using whole-exome sequencing. Exp. Ther. Med. 20, 1447–1454. doi: 10.3892/etm.2020.8890 32742378 PMC7388290

[B163] Watanabe M. Houten S. M. Mataki C. Christoffolete M. A. Kim B. W. Sato H. . (2006). Bile acids induce energy expenditure by promoting intracellular thyroid hormone activation. Nature 439, 484–489. doi: 10.1038/nature04330 16400329

[B164] Watanabe T. Yamamura T. Watanabe M. Yasuo S. Nakao N. Dawson A. . (2007). Hypothalamic expression of thyroid hormone-activating and -inactivating enzyme genes in relation to photorefractoriness in birds and mammals. Am. J. Physiol. Regul. Integr. Comp. Physiol. 292, R568–R572. doi: 10.1152/ajpregu.00521.2006 17197645

[B165] Wouters H. J. van Loon H. C. van der Klauw M. M. Elderson M. F. Slagter S. N. Kobold A. M. . (2017). No effect of the Thr92Ala polymorphism of deiodinase-2 on thyroid hormone parameters, health-related quality of life, and cognitive functioning in a large population-based cohort study. Thyroid 27, 147–155. doi: 10.1089/thy.2016.0199 27786042

[B166] Wu Z. Martinez M. E. St Germain D. L. Hernandez A. (2017). Type 3 deiodinase role on central thyroid hormone action affects the leptin-melanocortin system and circadian activity. Endocrinology 158, 419–430. doi: 10.1210/en.2016-1680 27911598 PMC5413080

[B167] Yamamoto T. Nakahata Y. Soma H. Akashi M. Mamine T. Takumi T. (2004). Transcriptional oscillation of canonical clock genes in mouse peripheral tissues. BMC Mol. Biol. 5, 18. doi: 10.1186/1471-2199-5-18 15473909 PMC535906

